# Real-Time Urban Traffic Monitoring System Based on Edge Computing: Vehicle Speed Estimation via Homography and Automatic License Plate Recognition on Jetson Orin NX for Andean Urban Corridors

**DOI:** 10.3390/s26216845

**Published:** 2026-10-29

**Authors:** Javier Mendoza-Calderon, Hemerson Lizarbe-Alarcon, Angel Hugo Vilchez-Peña, Diego O. Tenorio-Huarancca, Saul Walter Retamozo-Fernandez, Harby Pareja Melgar, Wilmer Moncada, Cristhian Aldana, Yesenia Saavedra, Edwin Saavedra, Carlos Carrasco-Badajoz, Carolina Rayme-Chalco, Renato Soca-Flores, Youll Bautista-Arroyo

**Affiliations:** 1 GECOTRAN Research Group (Studies in Management, Construction and Transport), Faculty of Mining, Geological and Civil Engineering, Universidad Nacional de San Cristóbal de Huamanga, Ayacucho 05000, Peru; javier.mendoza.16@unsch.edu.pe (J.M.-C.); hemerson.lizarbe@unsch.edu.pe (H.L.-A.); angel.vilchez@unsch.edu.pe (A.H.V.-P.); saul.retamozo@unsch.edu.pe (S.W.R.-F.); harby.pareja.16@unsch.edu.pe (H.P.-M.); 2 Remote Sensing and Renewable Energy Laboratory (LABTELER), Faculty of Mining, Geological and Civil Engineering, Universidad Nacional de San Cristóbal de Huamanga, Ayacucho 05000, Peru; wilmer.moncada@unsch.edu.pe; 3 Research Group on Productive Efficiency, Environment and Society (EPAS UNF), Universidad Nacional de Frontera, Sullana 20100, Peru; caldana@unf.edu.pe (C.A.); ysaavedra@unf.edu.pe (Y.S.); 4 Universidad Nacional de Frontera, Sullana 20100, Peru; esaavedran@unf.edu.pe; 5 Biodiversity and Geographic Information Systems Laboratory (BioSIG), Faculty of Mining, Geological and Civil Engineering, Universidad Nacional de San Cristóbal de Huamanga, Ayacucho 05000, Peru; carlos.carrasco@unsch.edu.pe (C.C.-B.); carolina.rayme@unsch.edu.pe (C.R.-C.); 6 Research Group on Environmental Physics, Remote Sensing, and Instrumentation (FATI), Faculty of Mining, Geological and Civil Engineering, Universidad Nacional de San Cristóbal de Huamanga, Ayacucho 05000, Peru; renato.soca@unsch.edu.pe (R.S.-F.); youll.bautista@unsch.edu.pe (Y.B.-A.)

**Keywords:** vehicle speed estimation, license plate recognition, edge computing, YOLO26, homography, Kalman filter, ByteTrack, Jetson Orin NX, traffic enforcement, smart city

## Abstract

This paper presents a distributed edge computing system for real-time urban traffic monitoring that integrates vehicle speed estimation and automatic license plate recognition (ALPR) on NVIDIA Jetson Orin NX devices. The system addresses the need for low-cost, automated traffic enforcement in developing urban environments where certified radar-based speed measurement infrastructure is not available. The proposed architecture consists of two independent processing nodes. The first node performs speed estimation using an IMOU aerial camera, YOLO26n-based vehicle detection, ByteTrack multi-object tracking, perspective homography transformation, and Kalman filtering for instantaneous speed computation. The second node performs license plate recognition employing a frontal camera, YOLO26n detection, and a Compact Convolutional Transformer (CCT) OCR model for real-time plate reading. Both subsystems are deployed on Jetson Orin NX modules with TensorRT FP16-optimized models, sustaining 20.0–22.3 FPS for speed estimation and 29–36 FPS for license plate recognition. The system incorporates the Peruvian traffic regulation (DS 025-2021-MTC) for automated violation classification, including speeding offenses (code M20) with the 5 km/h camera tolerance threshold introduced by DS 011-2026-MTC. Field validation was conducted at Jr. Carlos F. Vivanco, Ayacucho, Peru (2719–2725 m a.s.l.). Speed estimation was validated in a 40-pass nocturnal campaign against 1 Hz GNSS Doppler velocity spanning 16.9–48.8 km/h, a range that covers both the 30 km/h statutory limit and the 35 km/h enforcement tolerance. The configuration deployed during the campaign, which smooths the metric foot-point with a constant-velocity Kalman filter, attained a mean absolute error of 3.19 km/h at the enforcement line. The residual was identified as the steady-state lag of that motion model under vehicle acceleration, with which it correlates at *r* = −0.727; replacing it with a constant-acceleration model reduces the error to 1.32 km/h under leave-one-pass-out cross-validation and removes the dependence on acceleration entirely (*r* = +0.003). The improvement was verified to transfer to the Jetson deployment node. Two earlier campaigns are retained for completeness because they identified deployment failure modes—an RTSP stream resolution renegotiation and a speed-display defect affecting vehicles stopped beyond the measurement zone—that are reported as engineering contributions in their own right. A comprehensive model development study, comprising six training experiments (M1–M6) across three architectures (YOLOv8m-seg, YOLO11m-seg, YOLO26-seg/det), documents the methodological transition from instance segmentation to bounding-box detection driven by edge computing latency constraints. The results demonstrate the feasibility of deploying a traffic enforcement prototype using commercial edge devices and IP camera infrastructure, offering a scalable and cost-effective solution for smart city applications in developing regions.

## 1. Introduction

Excessive vehicular speed is the most significant modifiable risk factor in urban traffic crashes: a 5% reduction in average speed produces a 30% decrease in the probability of a fatal collision [1]. A recent systematic review of 94 studies confirms that speed cameras reliably reduce driving speeds, although the magnitude of the effect depends on road type, speed limit, and the geometry of camera placement [2]. Yet enforcement coverage remains critically insufficient across low- and middle-income countries [3]. In Peru, the National Road Safety Observatory (ONSV) recorded 87,083 traffic crashes in 2023, with speeding identified as the main contributing factor in 26.4% of cases [4]. The Ayacucho region illustrates the local severity of this problem: 549 crashes were recorded in 2024, a 32.3% increase over the previous year, and 49.1% of fatal crashes between 2021 and 2023 were concentrated in Huamanga province [5,6].

Enforcing posted speed limits requires certified measurement equipment, and this is where the gap becomes structural rather than incidental. Traditional enforcement relies on radar-certified cinemometers or inductive loop detectors, whose acquisition, installation, and maintenance costs place them out of reach for most municipal budgets. SUTRAN, Peru’s national land transport authority, concentrates its certified cinemometer enforcement on a limited set of national highway segments with high accident rates; its published monthly enforcement schedules do not extend to the urban corridors of medium-sized Andean cities such as Ayacucho. The calibration and homologation requirements of the Peruvian metrological standard (NMP 013:2010) add further cost and administrative burden, effectively excluding municipal governments from deploying certified enforcement infrastructure on their own. Even in Lima, the capital and by far the best-resourced municipality in the country, mobile-cinemometer enforcement only began on a limited, gradually expanding schedule, underscoring that the barrier is structural rather than a matter of city size alone. The result is a regulatory gap with direct consequences: speed limits exist on paper but go unmonitored in practice.

Vision-based speed estimation and edge-based automatic license plate recognition (ALPR) offer a cost-effective alternative, reusing surveillance cameras already deployed in urban environments and performing inference locally on embedded GPU platforms such as the NVIDIA Jetson family, without reliance on cloud connectivity. Speed estimation methods fall broadly into virtual line crossing, which derives speed from the transit time between two reference lines [7,8,9], and homography-based transformation, which maps pixel displacements to metric road-plane coordinates and provides instantaneous speed throughout the calibrated zone [10,11,12,13]. ALPR has progressively collapsed its classical four-stage pipeline [14] into end-to-end deep learning models [15,16], with temporal aggregation across frames improving robustness to motion blur and partial occlusion [17,18]; illumination variation, multi-angle tilt, and region-specific plate formats remain open challenges [19]. On embedded GPU platforms, prior work has demonstrated the feasibility of edge-based ALPR and speed detection individually [20,21,22], including recent work connecting video monitoring to large-scale vehicle-trajectory data [23,24], but, to the best of our knowledge, no published system combines speed estimation and ALPR on a single distributed edge deployment with automated violation classification against a national regulatory framework; Section 2 reviews this literature in detail.

The present work extends a research program on intelligent traffic management in Andean historic urban centers, which has previously addressed traffic-light optimization [25], traffic-sign recognition [26], and vulnerable-road-user detection [27] at the same study site. This study completes that research line by addressing the remaining enforcement gap: automated speed measurement and license plate reading on edge devices, with violation classification aligned with Peruvian traffic regulation.

Based on the literature reviewed in Section 2, this work addresses three gaps. First, to the best of our knowledge, no published system integrates homography-based speed estimation and ALPR on TensorRT-optimized, distributed edge nodes with automated violation classification validated against a national regulatory framework, particularly in a developing-country urban context. Second, the combination of homography-based speed estimation with virtual line crossing as a mutual cross-validation strategy has not been reported for congested historic urban corridors, where single-method approaches are known to fail. Third, the Compact Convolutional Transformer (CCT) architecture for license-plate OCR has not been benchmarked against established alternatives such as EasyOCR under real-time edge deployment conditions.

This article makes four main contributions, each tied to a quantitative result reported in Section 4. First, a field-deployed, two-node edge computing system (two NVIDIA Jetson Orin NX units connected through a local Ethernet network) that performs simultaneous vehicle speed estimation and automatic license plate recognition and automatically classifies speed violations under Peruvian regulation DS 025-2021-MTC as updated by DS 011-2026-MTC, operated at an active municipal installation rather than evaluated in a laboratory or simulation. Second, a dual-method speed estimation pipeline, combining homography-based transformation with Kalman filtering and cross-validated against virtual line crossing, calibrated through drone orthophotography and topographic surveying, and validated in a 40-pass nocturnal campaign against GNSS Doppler reference velocity (mean absolute error of 1.32 km/h after correcting a motion-model bias identified during validation). Third, a license-plate recognition pipeline (YOLO26n detection with a Compact Convolutional Transformer OCR model) benchmarked against EasyOCR on the Peruvian plate format. Fourth, a transparent account of the full engineering development process, including a six-experiment model comparison (M1–M6 across three architectures) that documents a negative result for instance segmentation under edge-latency constraints, and the field diagnosis and correction of two previously unreported deployment failure modes—an RTSP stream resolution renegotiation and a speed-display defect—together with a characterization of hardware import costs as a structural barrier to replication in the Peruvian context.

The remainder of this paper is organized as follows. Section 2 reviews related work on vision-based speed estimation, automatic license plate recognition, and edge computing deployments. Section 3 describes the study site, hardware architecture, and methods. Section 4 presents the results of model development, field validation, and license plate recognition evaluation. Section 5 discusses the main findings and their implications. Section 6 concludes the paper and outlines directions for future work.

## 2. Related Work

The literature reviewed below was compiled from the authors’ existing collection of primary sources on vision-based traffic monitoring, vehicle speed estimation, and automatic license plate recognition, built up over the course of this research program and maintained in a Zotero reference library. Each citation was cross-checked against that library’s bibliographic record (author names, venue, year, and, where applicable, DOI) prior to inclusion, specifically to prevent the citation inaccuracies that an unverified literature pass can introduce. This review does not follow a systematic multi-database search protocol with a fixed search string or screening procedure; it is best described as a broad, author-curated survey of the directly relevant literature, supplemented during peer review by targeted searches for specific gaps identified by reviewers.

### 2.1. Vision-Based Vehicle Speed Estimation

The estimation of vehicle speed from monocular video streams constitutes a well-established research domain within intelligent transportation systems. Dilek and Dener surveyed the breadth of computer vision applications in ITS, identifying speed estimation as one of the critical capabilities enabled by deep learning advances [28]. Lv et al. complemented this perspective with a focused review of YOLO algorithm applications across traffic monitoring, vehicle detection, and sign recognition [29]. Ali and Zhang traced the architectural evolution of the YOLO framework from its inception through YOLO11, benchmarking detection accuracy and inference speed across successive versions [30].

Virtual line crossing represents the most widely adopted speed estimation paradigm. Lin et al. combined YOLOv4 with a Gaussian mixture model, achieving a 7.6% average speed error [8]. Merrad et al. deployed YOLOv7 with DeepSORT, reporting less than 3% error [7]. Islam et al. extended the approach with IoT integration and ByteTrack tracking, attaining an MAE of 3.2 km/h [31]. However, all line-crossing methods share a critical limitation: they cannot register speeds when vehicles stop between the reference lines. The present work overcomes this limitation through a dual-method approach in which homography-based instantaneous measurement operates continuously regardless of vehicle stops, while line crossing serves as cross-validation when vehicles complete the traversal.

Homography-based methods address the line-crossing limitation. Rodríguez-Rangel et al. achieved an MAE of 1.694 km/h using YOLOv3 and Kalman filtering [10]. Ahmed et al. demonstrated viability from UAV platforms with errors as low as 0.53 km/h [11]. Lian et al. reported 97.6% average accuracy with monocular cameras [12]. Sangsuwan and Ekpanyapong achieved an MAE of 3.38 km/h validated against a laser reference [32]. Kamil et al. paired a learned homography network with YOLOv8 and ByteTrack, validating against a speed-gun reference across 2408 vehicles [33]. Macko et al. replaced RetinaNet with YOLOv6 in a vanishing-point-based 3D bounding-box method, reporting a 0.58 km/h median error on BrnoCompSpeed at eight times the prior inference speed, with explicit evaluation on edge hardware [34]. Nevertheless, these systems operate on highways or controlled environments with free-flowing traffic. None has been validated in congested historic city centers with narrow colonial streets, descending slopes, and heterogeneous vehicle fleets including mototaxis, which represent the specific conditions addressed by the present system.

End-to-end approaches have emerged recently. Kesli and Kilicarslan proposed Speed-YOLOv8, attaining an MAE of 1.34 km/h on the UTFPR dataset [35]. García-Aguilar et al. estimated speed from apparent object size [36]. Marikhu et al. achieved a 1.62 km/h mean error using optical flow [37]. Although these methods are computationally elegant, they require dataset-specific training that limits their transferability. The present work employs explicit geometric calibration through drone orthophotography, enabling deployment at any new site through a repeatable topographic survey procedure without model retraining.

Hybrid approaches include HVD-Net by Ashraf et al. (87.2% accuracy) [38], centroid-based tracking by Vyas and Patel [9], and CNN-based methods by Mishra et al. [39]. Wang et al. reconstructed 3D vehicle parameters for safety analysis [40]. None of these systems integrates speed estimation with license plate recognition on edge hardware, nor implements automated violation classification against a specific national regulatory framework, two capabilities central to the present contribution.

Nighttime operation poses distinct challenges. Lashkov et al. addressed low-illumination sensing through headlight-based detection [41] and GMM clustering [42]. Swathi et al. evaluated detection under variable lighting [43]. The present system extends nighttime capability through TensorRT-optimized inference on Jetson Orin NX, with field validation under both daytime and nighttime conditions demonstrating sustained detection performance, although with acknowledged degradation in vehicle type classification accuracy at night due to contour loss in dark-colored vehicles.

### 2.2. Automatic License Plate Recognition

The ALPR field has evolved from traditional four-stage pipelines [14] toward progressively integrated deep learning architectures. Wang and Tu surveyed methods for complex environments [44]. Xu et al. provided the most recent comprehensive review, identifying cross-regional generalization as a critical open problem [19]. The present work contributes to this open problem by evaluating ALPR on Peruvian license plates, a format absent from all reviewed benchmarks, under the specific visual conditions of high-altitude Andean urban environments with colonial architecture.

Detection-stage advances have been driven by YOLO. Laroca et al. introduced the UFPR-ALPR benchmark [15]; the same group’s more recent work benchmarked 16 OCR models across 12 public datasets and found that synthetic-real data fusion substantially improves cross-dataset generalization, a persistent weakness in multinational ALPR [45]. Satya et al. optimized YOLOv8 for resource-constrained devices at 30 FPS [46]. Wang et al. proposed VSNet, surpassing 99% accuracy at 149 FPS [47]. The present system employs YOLO26n, a more recent and lightweight architecture than those evaluated in the cited works, optimized with TensorRT for Jetson Orin NX, achieving 29–36 FPS for simultaneous vehicle detection and plate localization.

Recognition approaches diverge between paradigms. Gonçalves et al. proposed joint detection and recognition [48]. Silva and Jung developed a two-pass CNN for Brazilian plates [49]. Ke et al. employed lightweight MRNet [16]. Pham proposed networks for CPU devices [50]. Unlike these approaches that use custom recognition networks requiring extensive training data, the present system employs a pre-trained Compact Convolutional Transformer (CCT) model of only 5 MB, achieving 43.1 ms per plate on CPU without competing for GPU resources with the detection model.

Temporal information exploitation has emerged as a key research direction. Yoo and Han introduced temporal matching [17]. Che et al. proposed character time-series aggregation [18]. Quiala et al. evaluated frame combination strategies [51]. Na et al. addressed multi-frame restoration [52]. Wang et al. constructed the LSV-LP benchmark [53]. The present system implements an analogous temporal strategy through ByteTrack persistent tracking with periodic OCR re-reading (every 3 frames per tracked plate), consolidating multiple readings per vehicle to reduce character-level errors.

Edge deployment has received growing attention. Ammar et al. achieved 17.1 FPS on Jetson Xavier AGX [20], the closest comparable system to the present work. Sonnara et al. proposed Light-Edge, a single-pass ResNet-18+FPN network sustaining 14 FPS on a 10 W Jetson Nano budget, 73% faster than an anchor-free baseline while halving power consumption [54]; the present work targets the more capable Jetson Orin NX and reports 29–36 FPS for the plate-recognition node accordingly. However, their architecture processes speed and plate recognition on a single node, whereas the present system distributes these tasks across two independent Jetson Orin NX nodes, eliminating GPU contention between detection models and achieving a higher aggregate throughput (20.0–22.3 FPS + 29–36 FPS simultaneously). Netinant et al. evaluated Raspberry Pi deployment [55]. Meesad and Thumthong achieved 99.16% on Jetson Nano for Thai plates [21]. Ashrafee et al. addressed Bangla plates [56]. Viswanathan et al. proposed the FANVID low-resolution benchmark [57]. Ribeiro and Hirata proposed Visual Rhythm [58]. Region-specific studies include Philippine plates [59], Indian plates [60], theft detection [61], and vision-language models [62]. To the best of the authors’ knowledge, very few published ALPR studies have addressed Peruvian license plate formats under real-world Andean urban conditions.

Beyond enforcement, LPR data has become a central source in transportation research for reconstructing full-network trajectories from sparse camera observations. Li et al. formulated this as a Markov decision process solved via inverse reinforcement learning [23], and Hu et al. used the resulting reconstructed paths to estimate Macroscopic Fundamental Diagram parameters across regionalized sub-networks [63]. He et al. applied a Bayesian deep learning approach to reconstruct lane-based arrival curves at signalized intersections directly from LPR readings [64]. Ng et al. surveyed this body of work from 2006 to the present, documenting its role in estimating and predicting traffic state, analyzing travel demand, optimizing traffic control, and enhancing traffic safety management [65]. Drone-based swarms offer a complementary, spatially complete alternative: Barmpounakis and Geroliminis’s pNEUMA experiment recorded close to half a million trajectories over central Athens [24], and Fonod et al.’s continuation at Songdo, South Korea, produced approximately 700,000 georeferenced vehicle trajectories across 20 intersections [66]; Chen et al. similarly released a dense-conflict trajectory dataset for signalized intersections [67]. These campaigns provide complete but episodic coverage, in contrast to the continuous, low-cost observation that fixed LPR cameras such as the present system’s can sustain indefinitely once deployed. The present prototype contributes to this line of work precisely as these studies assume LPR data to be available: time-stamped plate reads paired with a simultaneous, per-vehicle speed measurement, rather than plate reads alone.

### 2.3. Integrated Traffic Enforcement Systems

The integration of speed estimation with license plate recognition into unified enforcement systems remains comparatively underexplored. The majority of published works address these capabilities in isolation. Speed estimation systems measure velocity but do not identify vehicle license plates [10,12,35], while ALPR systems read plates but do not measure speed [16,20,21]. The few integrated approaches reported in the literature rely on cloud processing or certified radar. Villa et al. reported the closest integrated architecture, performing vehicle detection, classification, tracking, speed estimation, and geolocation on edge hardware without cloud dependency [68]; their system, however, does not read license plates or classify violations against a specific regulatory framework, the two capabilities the present system adds.

Table 1 synthesizes the comparative positioning of the present work against the most relevant systems in the literature. The present work is the only system that simultaneously provides dual-method speed estimation (homography plus line crossing) for congested environments, ALPR with lightweight CCT OCR on a separate edge node, automated violation classification against a national regulatory framework, and field validation under real traffic conditions in a developing-country historic city center.

## 3. Materials and Methods

### 3.1. Study Area and Topographic Survey

The study was conducted on Jr. Carlos F. Vivanco, a narrow colonial-era street located in the historic center of Ayacucho (Huamanga), Peru, at an altitude between 2719 and 2725 m a.s.l. (Figure 1). This street serves as a principal commercial corridor connecting the historic core with the southern residential districts, channelling a heterogeneous mix of vehicles that includes automobiles, motorcycles, microbuses, mototaxis, pickup trucks, combis, and cargo trucks through a single traffic lane approximately 6 m wide. The site was selected for three main reasons. First, it constitutes the same intersection studied by Tenorio-Huarancca et al. [25], which enables direct methodological continuity. Second, the street exhibits a measurable descending slope that introduces a non-trivial geometric correction requirement for speed estimation. Third, the traffic conditions, which include frequent congestion, mixed vehicle types, colonial architecture shadows, and a fixed-cycle traffic light at the downstream intersection, represent the typical operational challenges of Andean historic urban corridors.

The topographic characterization of the study segment was conducted through three independent measurement methods to ensure metric reliability. First, a tape measure was used for direct field measurement along the inclined road surface, yielding an approximate distance of 92–93 m with an estimated precision of ±10–20 cm. Second, the horizontal distance was measured in Google Earth Pro, obtaining 92.9 m with an estimated precision of ±1–3 m. Third, a drone-based orthophotographic survey was performed to generate a georeferenced orthomosaic, which was processed in Autodesk AutoCAD Civil 3D 2025 to obtain a calibrated horizontal distance of 84.1 m between the two primary reference points (L1 and L2) with an estimated precision of ±10 cm. The discrepancy between the total segment length (92.9 m) and the L1–L2 distance (84.1 m) reflects that the reference points do not coincide with the segment endpoints: L1 is located at the reference post and L2 at the pavement slab, both situated within the broader monitored corridor. Table 2 summarizes the three measurement approaches.

The elevation profile obtained from Google Earth Pro (Figure 2) reveals a descending slope of 5.94% along the study segment, with an elevation change of −5.52 m over 92.9 m (from 2725 to 2719 m a.s.l.). The corresponding slope correction factor is calculated as $f_{s}=1/\cos\left(\arctan\left(0.0594\right)\right)=1.002$, representing a 0.18% correction that converts a horizontal distance to the true inclined p ath length traveled by the vehicle. A second, independent correction arises because both the homography and the line-crossing methods derive their metric distances from UTM projected coordinates: a grid-to-ground factor of 0.074% applies at the study site’s mean elevation of 2722 m a.s.l. and its distance from the Zone 18S central meridian, converting UTM grid distances to true horizontal ground distances. Combined, $f_{s}=1.002\times 1.00074\approx 1.0025$ (approximately 0.25%). Since both the homography-based and line-crossing methods ultimately compute displacement from UTM-derived coordinates, both corrections apply to both methods, as detailed in Section 3.2.3. At 30 km/h, the combined correction amounts to 0.075 km/h, which is negligible compared to the system measurement uncertainty, but is included for methodological rigor.

### 3.2. Homography Calibration

The perspective homography matrix H establishes a projective mapping between pixel coordinates in the camera image plane and metric coordinates on the road surface plane. This calibration is the geometric foundation of the instantaneous speed estimation method, enabling the conversion of frame-to-frame pixel displacements into real-world distances in meters.

#### 3.2.1. Drone Orthophotographic Survey

A drone-based aerial survey was conducted over the study segment to generate a high-resolution orthomosaic serving as the metric reference for the calibration. The resulting orthophoto possesses centimeter-level spatial resolution, allowing the precise identification of pavement and curb features used as geometric control points. The orthophoto was imported into Autodesk AutoCAD Civil 3D 2025 for geometric processing (Figure 3). Two primary reference lines were established on the orthophoto: L1 at the base of a reference post (corresponding to pixel row y=94 in the camera image) and L2 at a distinctive pavement slab (corresponding to pixel row y=665). The horizontal distance between L1 and L2, measured with the AutoCAD dimension tool, is 84.1 m. This distance serves a dual purpose: it defines the spatial extent of the speed measurement zone, and it provides the known distance for the virtual line-crossing speed estimation method.

**Figure 3:**
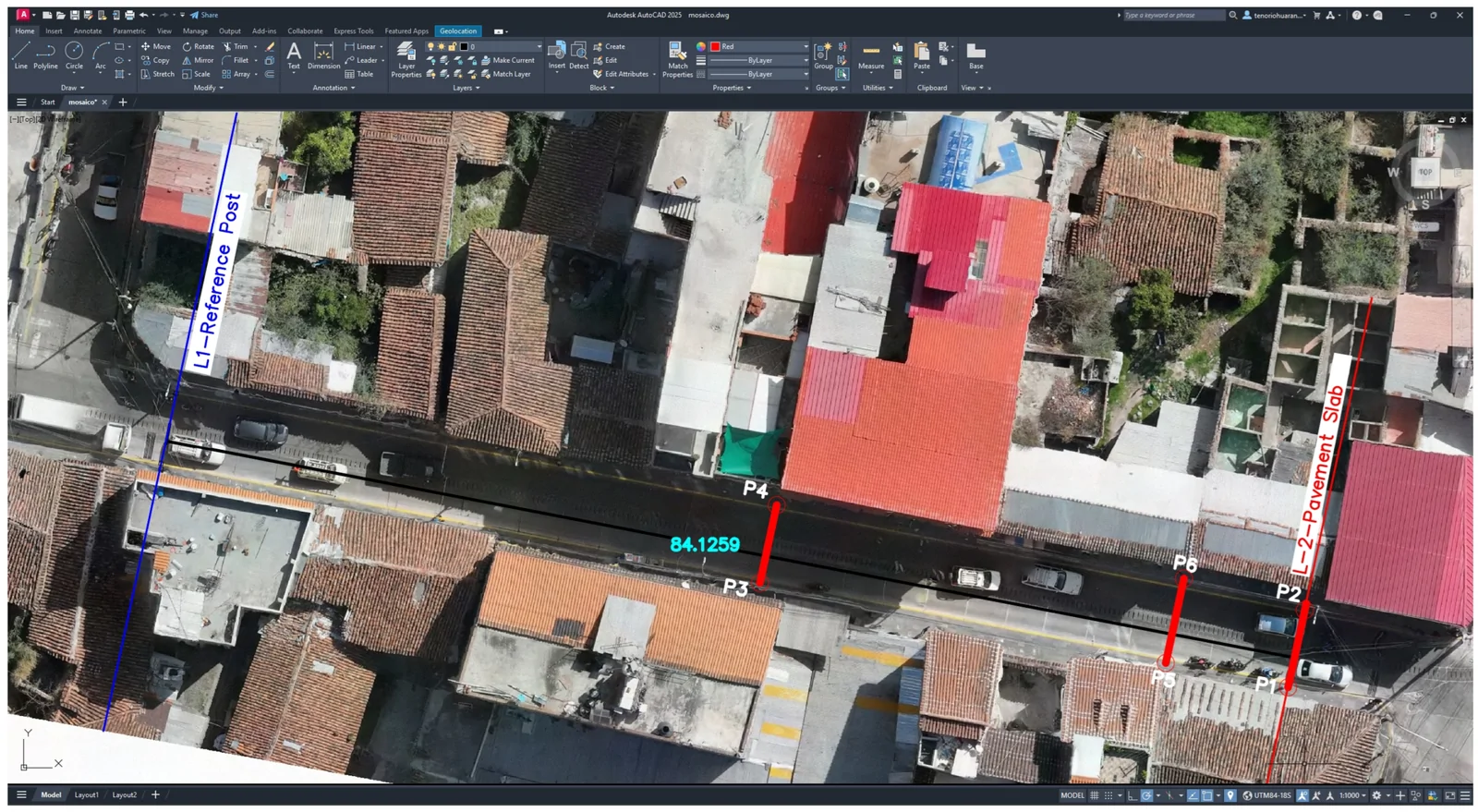
Drone orthophoto processed in Autodesk AutoCAD Civil 3D 2025 showing the calibrated study segment. L1 (Reference Post) and L2 (Pavement Slab) delimit the 84.1 m measurement zone. Six homography calibration points (P1–P6) are identified at ground-level features along the curb-road junctions on both sides of the carriageway. Point coordinates are listed in Table 3.

**Table 3:** Homography calibration point coordinates. Pixel coordinates (u,v) are referenced to the IMOU camera frame (1280 × 720 px). Metric coordinates (Easting, Northing) correspond to UTM Zone 18S (WGS84), obtained from the drone orthophoto by means of AutoCAD Civil 3D 2025.

Point	*u* (px)	*v* (px)	Easting (m)	Northing (m)	Physical Feature
P1	244	658	584,111.777	8,544,615.739	Curb-road junction, left, near (L2)
P2	585	665	584,112.827	8,544,620.703	Curb-road junction, right, near (L2)
P3	483	142	584,073.068	8,544,623.187	Post base, far zone (L1)
P4	587	142	584,074.171	8,544,628.312	Post base, far zone (L1)
P5	358	420	584,102.804	8,544,617.465	Curb-road junction, left, mid-depth
P6	583	420	584,103.944	8,544,622.761	Curb-road junction, right, mid-depth

#### 3.2.2. Calibration Point Selection

Six ground-level calibration points were selected at identifiable features along the curb-road junction, ensuring that all points lie on the road surface plane as required by the planar homography assumption. The points were distributed across three depth zones within the camera field of view to maximize the geometric coverage of the homography: two points near the camera (P1, P2 in the L2 zone, v≈660 px), two at intermediate depth (P5, P6, v=420 px), and two in the far zone (P3, P4 in the L1 zone, v=142 px). Within each depth zone, the points were placed on opposite sides of the carriageway (left and right curb) to provide lateral separation.

For each calibration point, two coordinate pairs were obtained: metric coordinates in the UTM reference system (Zone 18S, WGS84) read from the drone orthophoto using the AutoCAD ID command, and pixel coordinates (u,v) identified on a reference frame captured from the IMOU aerial camera (1280 × 720 px) using a superimposed coordinate grid (Figure 4). Table 3 lists the complete coordinate pairs for the six calibration points.

#### 3.2.3. Homography Matrix Computation

The 3×3 homography matrix H was computed using OpenCV’s cv2.findHomography function with the ordinary least-squares method (method=0) using all six calibration points. The use of six points, exceeding the four-point minimum required by cv2.getPerspectiveTransform, provides an overdetermined system solved by least-squares, yielding a well-conditioned estimate and a meaningful reprojection error metric across all six correspondences. The homography maps pixel coordinates (u,v) to local metric coordinates $\left(x_{m},y_{m}\right)$ on the road surface:

(1) $$\left[\begin{array}{c} x^{\prime }\\ y^{\prime }\\ w^{\prime } \end{array}\right]=H\left[\begin{array}{c} u\\ v\\ 1 \end{array}\right],\;x_{m}=\frac{x^{\prime }}{w^{\prime }},\;y_{m}=\frac{y^{\prime }}{w^{\prime }}$$

where ${\left(u,v,1\right)}^{T}$ is the pixel coordinate in homogeneous form, ${\left(x^{\prime },y^{\prime },w^{\prime }\right)}^{T}$ is the resulting homogeneous vector, and the metric coordinates $\left(x_{m},y_{m}\right)$ are recovered by dividing through by the scale component $w^{\prime }$, as required for a projective (non-affine) transformation. The local metric coordinate system is defined by subtracting the UTM coordinates of calibration point P1 from all six points’ metric coordinates prior to fitting H, yielding a local origin at P1 (local coordinates (0,0)) that avoids the numerical precision issues associated with large UTM values; this is the origin convention used throughout Equation (7).

The quality of the homography calibration was assessed through the reprojection error, computed for each of the six calibration points by transforming its pixel coordinates through H and measuring the Euclidean distance to the corresponding ground-truth metric coordinate. This is the appropriate validation metric for H, since it is computed strictly within the span of the calibration points used to fit the transformation (approximately 0–39.4 m along the longitudinal axis, extended to a conservative reliable zone of 0–42 m). The L1–L2 distance of 84.1 m, measured independently from the drone orthophoto, is not used to validate H: L1 lies beyond the farthest calibration points (P3, P4, at pixel row y=142) and thus outside the region in which the homography was calibrated, so transforming it through H would extrapolate the model rather than validate it. The 84.1 m distance instead serves exclusively as the known separation for the complementary line-crossing speed estimation method, which operates independently of the homography.

An important methodological clarification concerns the application of the slope and grid-to-ground correction factors. The homography matrix H is calibrated using UTM Easting/Northing coordinates (Section 3.1), which are horizontal projected coordinates and therefore do not encode the terrain elevation of the calibration points. Consequently, displacements computed from H-transformed coordinates represent horizontal projected distances, not the true inclined distance traveled along the road surface, in exactly the same way as the line-crossing method’s horizontal reference distance. Both methods are therefore subject to the same combined correction factor $f_{s}=1.0025$ (Section 3.2.2), comprising the slope correction (0.18%) and the UTM grid-to-ground correction (0.074%) described above. This revises an earlier internal assumption that the homography inherently incorporated the terrain geometry; the present formulation applies the correction consistently to both methods, as reflected in Equations (3) and (5).

### 3.3. Dataset Construction

#### 3.3.1. Aerial Dataset

The aerial dataset was originally collected for the traffic light optimization study conducted at the same intersection by Tenorio-Huarancca et al. [25], and was reused in the present work to maintain methodological continuity. Video recordings were captured with the IMOU aerial camera under diverse environmental conditions including daylight, overcast, rain, dusk, night, and colonial architecture shadow interference.

The raw dataset comprised 2996 images with 2985 corresponding LabelMe JSON annotation files. Eleven images without annotations were discarded. All annotations consisted of instance-level segmentation polygons manually drawn in LabelMe, totaling 45,335 annotated instances. A critical discovery during preprocessing was that the dataset contained two mixed resolutions: 1892 images at 1920 × 1080 and 1093 images at 2688 × 1664. Polygon coordinate normalization was performed per-image using each JSON’s imageWidth and imageHeight fields, rather than hard-coded resolution values. This per-image approach prevented polygon corruption that would have affected 63.4% of the dataset (the 1892 images at 1920 × 1080) had the higher 2688 × 1664 resolution been incorrectly assumed as fixed for all images.

The original annotation schema included ten classes. During cleaning, two corrections were applied: the PERSON class (829 instances) was removed as being outside the vehicular scope of the system, and a single instance labeled as B (a typographical error) was corrected to MB. Additionally, 21 polygon vertices with coordinates exceeding image boundaries were clipped to valid ranges. The resulting clean dataset contains 2985 images with 44,505 vehicle instances across eight classes. Table 4 presents the class distribution.

The mototaxi class (MT) deserves particular attention: this three-wheeled motorized vehicle constitutes the primary informal transport mode in intermediate Andean cities, yet is completely absent from the established international vehicle detection datasets (COCO, BDD100K, KITTI). Its inclusion in the present dataset represents a contribution to the representation of developing-country vehicle taxonomies in computer vision research.

The dataset was partitioned using MultilabelStratifiedShuffleSplit from the iterative-stratification library [69] with seed 42 for reproducibility.

A binary multi-label vector [A,M,MB,PU,MT,CP,RC,CG] was constructed for each image, encoding the presence of each class. This multi-label stratification ensures proportional representation of all classes, including minority ones such as CG (241 instances), across the three partitions. The partition was performed in two stages: first, separating 10% as test, then splitting the remaining 90% into train (80%) and validation (10%), yielding 2384 training, 296 validation, and 305 test images. The validation and test sets were never modified after the partition.

The split is performed at the individual-image level and does not group images by capture session or by vehicle identity. The images were not extracted from continuous video at a fixed sampling interval; they were captured as discrete snapshots across separated time windows (peak-hour and off-peak periods on different days), which limits, without eliminating, the likelihood that two images separated by only a few seconds of the same vehicle were assigned to different partitions. No video-level or vehicle-level grouping constraint (e.g., GroupShuffleSplit keyed on capture session) was applied, so a residual risk of such near-duplicate frames crossing the train/test boundary cannot be ruled out; this is acknowledged as a limitation of the evaluation protocol (Section 5.3).

#### 3.3.2. Class Balancing via Ground-Aware Copy-Paste

The aerial dataset exhibits severe class imbalance, with an imbalance ratio (IR) of 77.5:1 between the most frequent class (A: 18,565) and the least frequent (CG: 241). To mitigate this imbalance, an offline augmentation strategy was applied exclusively to the training partition after splitting, ensuring zero data leakage into the validation and test sets.

An initial attempt using naive copy-paste augmentation, in which vehicle instances were pasted at random positions, produced unrealistic compositions: vehicles appeared on rooftops, sidewalks, and colonial mansion facades. This “unrealistic pasting context” artifact caused the model to learn spurious object-background associations, degrading rather than improving detection performance. The phenomenon is consistent with findings in the copy-paste augmentation literature [70].

Subsequently, a ground-aware copy-paste strategy was implemented, restricting vehicle placement to the road surface with perspective-consistent scaling and overlap avoidance against existing objects. The training set grew from 2384 to 8686 images, reducing the IR from 77.5:1 to approximately 29.2:1.

#### 3.3.3. Frontal Dataset for License Plate Recognition

The frontal dataset was constructed for the license plate recognition subsystem using two data sources.

Proprietary dataset (PLACAS_YOLO). A total of 1513 images were captured from the frontal camera position on Jr. Carlos F. Vivanco, annotated with eight classes (the same seven vehicle types plus a “plate” class for license plate regions). The dataset contains 10,368 instances across mixed resolutions (3840 × 2160 and 1920 × 1080), partitioned with the same MultilabelStratifiedShuffleSplit procedure (seed 42): 1210 training, 153 validation, and 150 test images.

*Combined dataset (DATASET_SUELO).* To evaluate the potential benefit of external data augmentation, the proprietary dataset (1513 images) was combined with four additional sources, detailed in Table 5. Not every available image was used from each source: the QHAWAY dataset [27] was filtered by class relevance, retaining only images containing at least one instance of the four vehicle classes shared with the present taxonomy (automobile, bus, truck, mototaxi) and discarding images that contained exclusively non-target classes (e.g., motorcycles, pedestrians); the *License Plate Recognition* dataset [71], an international multi-country collection of 10,125 images hosted on Roboflow Universe (CC BY 4.0), was randomly subsampled to a cap of 2000 images (seed 42, sampled proportionally across the train/validation/test splits) to prevent this single foreign-plate source from dominating the combined dataset; the two Peruvian-specific Roboflow datasets [72,73] were used in full, with a small number of images dropped for empty or invalid annotations after bounding-box-to-polygon conversion. The combined dataset totals 7555 images with 22,240 instances: 12,018 vehicle-class instances (7068 from the proprietary dataset, 4950 from QHAWAY) and 10,222 plate-class instances (3300 from the proprietary dataset plus 6922 contributed by the three Roboflow sources). Vehicle annotations derive exclusively from local Peruvian data, while external Roboflow sources contribute only plate-class instances, under the hypothesis that the rectangular shape and text-background contrast of license plates are universal features that generalize across countries.

A controlled comparison between the proprietary-only model (M6, trained on 1513 images) and the combined model (YOLO26n_suelo, trained on 7555 images) revealed that external plate data did not improve Peruvian plate detection performance (mAP50 of 0.815 vs. 0.807 for the plate class on the same 150-image test set). This negative result is reported as a methodological finding: the domain gap between international plate formats and Peruvian plates (six alphanumeric characters, specific font and dimensions) outweighs the benefit of additional training volume. The combined model was initially deployed under the assumption of improved performance; the post-hoc analysis demonstrates equivalence.

### 3.4. Model Training and Architecture Selection

#### 3.4.1. Training Configuration

All models were trained on a workstation equipped with an NVIDIA RTX 5080 GPU (16 GB VRAM) running Python 3.10 and Ultralytics 8.4.50. Deterministic training was enforced (seed=42, deterministic=True) to ensure reproducibility. Early stopping with a patience of 50 epochs was used for all experiments to prevent overfitting. The YOLO26 models employed the MuSGD optimizer (native to the YOLO26 architecture), while YOLOv8 and YOLO11 models used standard SGD.

Online data augmentation was applied with intensity that varied according to the training phase: Mosaic (1.0 → 0.5 → 0.0), MixUp (0.1 → 0.05 → 0.0), online copy-paste (0.1 → 0.05 → 0.0), HSV jitter (*h* = 0.015, *s* = 0.7, *v* = 0.4), and horizontal flip (0.5). The progressive reduction in augmentation intensity across training phases follows the principle that aggressive augmentation benefits early generalization, whereas minimal augmentation in later phases enables fine-tuning to the actual data distribution.

#### 3.4.2. Segmentation Models (M1–M4)

Four segmentation models were trained on the aerial dataset to establish a multi-architecture comparison and identify the optimal architecture for the target deployment.

M1 (YOLOv8m-seg): the 2023-generation medium-sized architecture, serving as the baseline. Trained at 640 px for 300 epochs with early stopping at epoch 124.

M2 (YOLO11m-seg): the 2024-generation medium architecture with architectural improvements over YOLOv8. Trained at 640 px with early stopping at epoch 123.

M3 (YOLO26m-seg): the 2025-generation architecture incorporating NMS-free inference, removal of Distribution Focal Loss (eliminating fixed bounding-box regression ranges), ProgLoss with Small-Target-Aware Label Assignment, and the MuSGD optimizer. Trained at 640 px with early stopping at epoch 204.

M4 (YOLO26m-seg, three-phase progressive): the same YOLO26m architecture trained with a progressive resolution strategy: Phase 1 at 640 px (240 epochs), Phase 2 at 800 px initialized from Phase 1 best weights (77 epochs), and Phase 3 at 1024 px initialized from Phase 2 best weights (21 epochs). Each subsequent phase inherits the learned representations from the previous phase while adapting to finer spatial details at higher resolution. The early convergence in Phases 2 and 3 (best epoch at 4 and 1, respectively) reflects the expected behavior of fine-tuning from a well-initialized checkpoint, rather than premature stopping.

A critical finding during M1 and M2 training was the identification of hyperparameter configurations that caused segmentation mask collapse: close_mosaic=10, mixup=0.1, copy_paste=0.1, lrf=0.01, and amp=True. These were corrected in M3 and M4 to close_mosaic=50, mixup=0.0, copy_paste=0.0, lrf=0.001, and amp=False, resolving the mask degradation and producing substantial improvements in segmentation metrics.

#### 3.4.3. Detection Models for Edge Deployment (M5–M6)

Despite M4 achieving the highest metrics among segmentation models (Box mAP50 of 0.920 on the test set), its deployment on the Jetson Orin NX produced only ∼5 FPS, well below the minimum threshold for real-time traffic monitoring. This performance bottleneck motivated the transition from instance segmentation to bounding-box detection using the nano-size architecture.

M5 (YOLO26n-det, aerial): trained on the balanced aerial dataset (8686 images) at 640 px with early stopping at epoch 81. The resulting model weighs 5.4 MB (10× smaller than M4) and achieves 20.2 FPS on the Jetson Orin NX with TensorRT FP16 optimization, a 4× acceleration factor over M4 at the cost of 6.4 percentage points in mAP50 (0.856 vs. 0.920). This trade-off is justified for the target application: a model operating at 20 FPS in real time provides continuous speed measurements, whereas a more accurate model at 5 FPS misses most vehicles in congested traffic.

M6 (YOLO26n-det, frontal): trained on the proprietary frontal dataset (1513 images, 8 classes including “plate”) at 640 px with early stopping at epoch 119. This model detects both vehicle types and plate regions, serving as the first stage of the ALPR pipeline.

Table 6 presents the comprehensive comparison of the six models on their respective test sets.

Table 7 details the per-class performance of M5 on the aerial test set, which constitutes the main detection model for the speed estimation subsystem.

The mototaxi class (MT) achieves the highest mAP50 (0.949) despite being a minority class (1286 instances), demonstrating that the ground-aware copy-paste augmentation and multi-label stratified partitioning effectively address class imbalance for this region-specific vehicle type. The rural combi class (RC) exhibits the lowest performance (mAP50 of 0.695), attributable to visual similarity with automobiles (confusion matrix analysis shows that 31% of RC instances are misclassified as A) and to the limited number of training instances (359 before augmentation). The consistency between validation and test metrics (mAP50 of 0.856 in both sets) confirms the absence of overfitting.

#### 3.4.4. Methodological Transition: From Segmentation to Detection

The progression from M1 to M5 documents a deliberate methodological evolution driven by the practical constraints of edge deployment. Segmentation models (M1–M4) were initially pursued because polygon-level annotations provide more accurate vehicle centroids for tracking and offer richer shape information for classification. The three-phase progressive training of M4 produced the best overall metrics (Box mAP50: 0.920, Mask: 0.891), validating the progressive resolution strategy.

However, deploying M4 on the Jetson Orin NX revealed that the computational overhead of mask prediction and rendering reduced the throughput to approximately 5 FPS, insufficient for real-time operation. The transition to M5 (YOLO26n-det) removed the segmentation head, reducing the model size from 54.5 MB to 5.4 MB and increasing throughput from ∼5 to 20.2 FPS. The bounding-box centroid (bottom-center point: $cx=(x_{1}+x_{2})/2$, $cy=y_{2}$) proved adequate for the homography-based speed estimation pipeline, since the vehicle contact point on the road surface is the coordinate relevant to the perspective transformation. This finding is consistent with the broader vision-based speed estimation literature, where bounding-box detection predominates over instance segmentation.

The complete training history, from initial segmentation experiments through hyperparameter correction, progressive resolution refinement, and final edge-optimized detection, is transparently reported to document the iterative nature of the model development process and to provide actionable guidance for researchers facing similar segmentation-versus-detection trade-offs in edge deployment scenarios.

### 3.5. System Architecture and Deployment

#### 3.5.1. Distributed Edge Computing Architecture

The system architecture follows a distributed edge computing paradigm with two nodes, where each processing task is assigned to a dedicated NVIDIA Jetson Orin NX device. This architectural decision was motivated by qualitative observations during preliminary single-device deployment testing: running both the vehicle detection model (M5) and the plate detection model (YOLO26n_suelo) concurrently on a single Jetson Orin NX produced visible GPU contention, with intermittent frame drops and unpredictable latency spikes that were not systematically benchmarked. A controlled, instrumented comparison of single-node versus two-node throughput, GPU utilization, and latency was not performed and is identified as a direction for future work; the two-node architecture reported here eliminated the observed resource contention and allowed each subsystem to operate at its sustained throughput of 20.0–22.3 FPS (speed estimation) and 29–36 FPS (license plate recognition), as reported in Section 4.

The complete hardware configuration is summarized in Table 8. Two Jetson Orin NX modules (16 GB LPDDR5, 157 TOPS INT8) serve as the computational core, each running a single inference pipeline. The speed estimation subsystem (Jetson .27) processes the aerial video stream from a fixed IMOU camera mounted approximately 9 m high on a metal bracket attached to a streetlight pole, capturing at 1280 × 720 px and 25 fps via RTSP over Ethernet. The license plate recognition subsystem (Jetson .28) processes the frontal video stream from a smartphone running the IP Webcam application at 800 × 600 px and 30 fps via MJPEG over WiFi 5 GHz. Both Jetson modules, the IMOU camera, and a monitoring display are interconnected through a WOW ATW-624G router providing four Gigabit Ethernet ports and dual-band WiFi (2.4/5 GHz). An Electrow 10.1-inch touchscreen display (1024 × 600 px) connected via DisplayPort to Jetson .27 runs Firefox in kiosk mode, displaying the live monitoring dashboard in the Serenazgo (municipal security) booth where the system is physically deployed.

#### 3.5.2. Network Configuration and Latency Optimization

The initial deployment used WiFi for all device connections, including the Jetson modules. This configuration produced round-trip latencies of 22–50 ms with high variance and intermittent micro-freezes in the video streams, caused by WiFi channel contention and signal attenuation inside the concrete Serenazgo booth. Migrating both Jetson modules and the IMOU camera to wired Ethernet connections reduced latencies to 0.75 ms (Jetson .27) and 0.88 ms (Jetson .28), representing a 30–65× improvement. The smartphone camera remains on WiFi as the only wireless device, which is acceptable given that the OCR pipeline tolerates higher latency without affecting plate reading accuracy. This migration from WiFi to Ethernet is reported as a deployment lesson: in enclosed concrete booths typical of municipal security installations, wired connections are essential for sustained real-time video processing.

#### 3.5.3. Speed Estimation Pipeline (Jetson .27)

The speed estimation node executes a sequential pipeline for each incoming frame comprising the following stages. First, frame acquisition from the IMOU camera via RTSP. Second, vehicle detection using the M5 model (YOLO26n-det, TensorRT [74] FP16 engine of 7.5 MB). Third, multi-object tracking with ByteTrack [75], using a 90-frame tracking buffer to maintain identity through brief occlusions. Fourth, extraction of the vehicle foot-point and homographic transformation to metric coordinates. Fifth, Kalman filtering for speed smoothing. Sixth, traffic violation classification against the MTC regulatory framework. Finally, MJPEG streaming through a Python ThreadingHTTPServer on port 8090. The pipeline sustains 20.0–22.3 FPS, sufficient for continuous speed monitoring at the native camera capture rate of 25 fps.

#### 3.5.4. License Plate Recognition Pipeline (Jetson .28)

The plate recognition node employs a multi-threaded architecture to decouple frame acquisition from inference, addressing a critical issue identified during development. OpenCV’s cv2.VideoCapture class, when configured to read from an HTTP MJPEG stream, maintains an internal buffer that accumulates frames faster than they can be processed. Calling cap.read() under this condition returns stale frames from the buffer instead of the most recent frame, causing the effective processing rate to collapse from 39.7 FPS to 0.6 FPS as buffer depth increases. This “buffer bloat” phenomenon rendered the system unusable for real-time plate reading, as vehicles would already have passed by the time their frames were processed.

The solution implements four concurrent threads. Thread 1 (Reader): runs a continuous cap.read() loop that discards all frames except the most recent, storing it in a shared variable protected by a threading lock. This thread runs at the native camera rate, continuously draining the OpenCV buffer. Thread 2 (Processor): retrieves the latest frame from the shared variable, performs vehicle detection with YOLO26n_suelo (TensorRT FP16 engine of 7.4 MB) and multi-object tracking with ByteTrack, extracts plate regions, and triggers OCR reading every 3 frames per tracked plate to balance accuracy against computational cost. Thread 3 (OCR): runs the Compact Convolutional Transformer (CCT) model (cct-s-v2-global-model, 5 MB ONNX) on CPU (device=’cpu’) for character recognition, deliberately avoiding GPU contention with the YOLO detection model running on Thread 2. Thread 4 (Server): serves the annotated video stream and monitoring dashboard via ThreadingHTTPServer on port 8091 in MJPEG format. The pipeline sustains 29–36 FPS, exceeding the real-time threshold.

Figure 5 presents the complete system architecture showing both processing nodes, data flows, and network connections. Figure 6 documents the corresponding physical deployment of the hardware in the field.

### 3.6. Speed Estimation

#### 3.6.1. Homography-Based Instantaneous Speed

The primary speed estimation method computes instantaneous vehicle speed at every frame by transforming pixel displacements into metric displacements through the calibrated homography matrix H (Section 3.2). For each tracked vehicle *i* at frame *t*, the foot-point (defined as the bottom-center point of the bounding box (cx,cy) where $cx=(x_{1}+x_{2})/2$ and $cy=y_{2}$) is extracted and transformed to metric coordinates:

(2) $$\left[\begin{array}{c} x^{\prime }\\ y^{\prime }\\ w^{\prime } \end{array}\right]=H\cdot \left[\begin{array}{c} cx^{\left(t\right)}\\ cy^{\left(t\right)}\\ 1 \end{array}\right],\;p_{m}^{\left(t\right)}=\left[\begin{array}{c} x^{\prime }/w^{\prime }\\ y^{\prime }/w^{\prime } \end{array}\right]$$

The raw instantaneous speed between consecutive frames is computed as:

(3) $$v_{\mathrm{raw}}^{\left(t\right)}=f_{s}\cdot \frac{∥p_{m}^{\left(t\right)}-p_{m}^{\left(t-1\right)}{∥}_{2}}{\Delta t}\times 3.6$$

where Δt=1/FPS, the factor 3.6 converts from m/s to km/h, and $f_{s}=1.0025$ is the combined slope and UTM grid-to-ground correction factor defined in Section 3.2.2. The Euclidean norm captures the total horizontal displacement on the road plane, accounting for both longitudinal and lateral vehicle motion, prior to correction to true ground distance. $f_{s}$ is defined here for methodological completeness and symmetry with the line-crossing method (Equation (5)), but is not applied in the deployed homography-based implementation: at the 30 km/h legal limit, its effect (0.075 km/h) is more than an order of magnitude below the 1.32 km/h mean absolute error of the best estimator characterized in Section 4.3.2, and comparable to the 18.3 cm mean reprojection error of the calibration itself, so its omission does not materially affect the reported speed estimates.

#### 3.6.2. Kalman Filter Smoothing

The raw footpoint position exhibits frame-to-frame fluctuations caused by bounding-box pixel variations, particularly for partially occluded vehicles or distant detections where small pixel errors translate to large metric errors through the homography. A two-dimensional constant-velocity Kalman filter [76] (own numpy implementation, independent of the filterpy package) is applied to the metric footpoint position of each tracked vehicle independently, with state vector $\left[x,y,v_{x},v_{y}\right]$ representing position and velocity in the local metric frame (Section 3.6) and acceleration modeled as process noise:

(4) $$x^{\left(t\right)}=\left[\begin{array}{c} x^{\left(t\right)}\\ y^{\left(t\right)}\\ v_{x}^{\left(t\right)}\\ v_{y}^{\left(t\right)} \end{array}\right],\;F=\left[\begin{array}{cccc} 1 & 0 & \Delta t & 0\\ 0 & 1 & 0 & \Delta t\\ 0 & 0 & 1 & 0\\ 0 & 0 & 0 & 1 \end{array}\right],\;H_{k}=\left[\begin{array}{cccc} 1 & 0 & 0 & 0\\ 0 & 1 & 0 & 0 \end{array}\right],\;v^{\left(t\right)}=\sqrt{{\left(v_{x}^{\left(t\right)}\right)}^{2}+{\left(v_{y}^{\left(t\right)}\right)}^{2}}$$

where $\left(x^{\left(t\right)},y^{\left(t\right)}\right)$ is the filtered footpoint position, $\left(v_{x}^{\left(t\right)},v_{y}^{\left(t\right)}\right)$ is the filtered velocity in the local metric frame, F is the state transition matrix, $H_{k}$ is the observation matrix (only position is measured; the subscript *k* distinguishes the Kalman observation matrix from the homography matrix H), and the reported speed $v^{\left(t\right)}$ is the Euclidean norm of the filtered velocity components, converted to km/h by the factor 3.6. The process noise covariance Q follows the standard discrete white-noise-acceleration model, block-assembled per axis from $\sigma_{a}$; the measurement noise covariance is $R=\sigma_{m}^{2}I_{2}$. The numerical values are: process noise standard deviation $\sigma_{a}=1.5$ m/s^2^ (representing expected acceleration variability), measurement noise standard deviation $\sigma_{m}=0.70$ m (representing homography-transformed position uncertainty), initial position covariance $P_{0,\mathrm{pos}}=1.0$ m^2^, initial velocity covariance $P_{0,\mathrm{vel}}=100.0$ (m/s)^2^ per axis (reflecting the zero-velocity initialization at track start), and a warmup period of 4 frames before the filtered output is used. These values were determined through iterative testing on a separate set of field recordings (not used in the validation reported in Section 4.3) by minimizing visible speed oscillation while preserving responsiveness to genuine acceleration events.

The Kalman filter serves two purposes: it suppresses high-frequency fluctuations from detection noise and provides a more physically plausible speed trajectory by imposing temporal continuity. Because the reported speed is recovered as the Euclidean norm of two filtered velocity components rather than from a scalar one-dimensional speed state, measurement noise on each axis is rectified (folded into a strictly non-negative magnitude) rather than averaging toward zero; this is the same mechanism discussed as Limitation 10 (Section 5.3) in connection with far-zone footpoint noise, and it means the filter does not, by construction, converge exactly to zero for a stationary vehicle. No explicit clamp of low-speed output to zero is implemented in the deployed code. The magnitude of the resulting floor was quantified in the September campaign (Section 4.3.2): across the paired readings for which the GNSS reference was below 2 km/h, the reported speed averaged 0.61 km/h with a median of 0.23 km/h and a maximum of 3.07 km/h, so the effect is real but sub-kilometre-per-hour in practice and does not prevent stops from being identified. The much larger non-zero plateaus observed in the field and reported in Section 4.3 were traced to a separate display defect and not to this mechanism. A one-dimensional scalar-speed filter, which would not exhibit norm rectification, remains a worthwhile refinement (Section 5.3).

#### 3.6.3. Complementary Line-Crossing Method

The virtual line-crossing method operates as a complementary measurement providing independent speed validation when vehicles traverse the full L1–L2 segment. Two horizontal detection lines are defined at pixel rows $y_{L1}=94$ (Reference Post) and $y_{L2}=665$ (Pavement Slab), corresponding to a calibrated horizontal distance of 84.1 m. When a tracked vehicle crosses both lines sequentially, the average speed is computed as:

(5) $$v_{\mathrm{line}}=\frac{84.1\times f_{s}}{t_{L2}-t_{L1}}\times 3.6$$

where $t_{L1}$ and $t_{L2}$ are the timestamps at which the vehicle foot-point crosses L1 and L2, respectively, and $f_{s}=1.0025$ is the combined slope and UTM grid-to-ground correction factor defined in Section 3.2.2. Unlike the homography-based method (Equation (3)), $f_{s}$ is applied in the deployed line-crossing implementation, using the value FACTOR_PENDIENTE = 1.0021 recorded in the inference code, an earlier calibration snapshot of the theoretical $f_{s}=1.0025$ derived in Section 3.2.2; the 0.04% relative difference between the two values is far below the system’s measurement uncertainty and was not corrected in the deployed code. Similarly, the deployed code stores the L1–L2 distance as DISTANCIA_M = 84.1259, the unrounded AutoCAD-read value; this additional precision is a software-storage artifact rather than an indication of sub-centimeter survey precision, which remains ±10 cm as reported in Table 2, consistent with the rounded 84.1 m figure used throughout the text.

The line-crossing method provides a single average speed per vehicle traversal, which serves as a cross-validation reference for the instantaneous homography-based measurements. Its primary limitation is that it cannot register speeds for vehicles that stop between L1 and L2 due to congestion or traffic light phases, a frequent occurrence at this site. This limitation motivated the development of the homography-based method as the primary measurement approach, while retaining line crossing for validation and as a secondary reference.

### 3.7. Temporal Alignment Between Video and GNSS Reference

Validating a video-derived speed against a GNSS reference requires the two time bases to be aligned. The aerial camera stamps each frame with its own real-time clock, which was not synchronised to any network time source, whereas the GNSS logger derives its timestamps from the satellite signal and is therefore referenced to UTC. Any offset between them maps directly onto the speed comparison whenever the vehicle is accelerating, and must be measured rather than assumed.

The offset was estimated from position rather than from speed. Both instruments measure the longitudinal coordinate *s* along the road axis independently—the proposed system through the homography, the logger through its GNSS fix—so aligning the two s(t) profiles uses an observable that is distinct from the quantity under validation. This matters because estimating the offset from the speed profiles would be circular: in a pass with monotonic acceleration, a temporal shift and a multiplicative speed bias produce the same effect on the residual and are not separately identifiable. Position does not have this degeneracy.

For each of the 40 passes, the shift minimising the RMSE between $s_{\mathrm{video}}\left(t+\delta \right)$ and $s_{\mathrm{GNSS}}\left(t\right)$ was found by grid search over δ∈[−6,+6] s. The estimates were tightly clustered at δ=+1.75 s (median; mean +1.59 s, SD 0.46 s, range +0.75 to +2.50 s), with no pass at the search boundary. Applying the correction reduced the position bias from −6.20 m to +0.98 m. The residual SD of 0.46 s is consistent with the 1 s quantisation of the burnt-in clock, which was read visually, combined with the 1 Hz GNSS sampling.

The offset was stable throughout the campaign: regressed against pass number it showed no trend ($r^{2}=0.001$; total drift 0.06 s over the 92 min of the campaign, equivalent to ≈0.9 s day^−1^). That figure is the expected order of magnitude for an uncompensated real-time clock, which independently supports attributing the offset to the camera clock rather than to the reference. Because the offset is a constant rather than a drift, a single correction applies to the whole campaign.

Two points are worth stating explicitly. First, the correction was derived without reference to the speed data, so it cannot have been tuned to improve the reported accuracy. Second, it does not improve it: after alignment the speed bias becomes marginally more negative (−3.38 to −3.54 km/h over all paired readings). The underestimation discussed in Section 4.3.2 is therefore a genuine property of the estimator and not an artefact of synchronisation. Results with and without the correction are reported in Table 9.

For future deployments we recommend synchronising the camera clock via NTP, which removes this source of uncertainty entirely.

### 3.8. Camera Displacement and Recalibration

Between the original calibration and the September campaign the aerial camera, mounted at approximately 9 m above the carriageway, shifted from its calibrated pose. The displacement was identified by re-checking the six control points against the reference frame, and the homography was re-fitted from a frame of the September recordings, yielding a control-point reprojection error of 31.1 cm (mean) and 45.3 cm (maximum).

We could not establish the cause with the evidence available, and do not claim one. A plausible mechanism, given the mounting height and the frequency of high winds and rainfall at the site, is progressive loosening of the mount under weather loading; this is offered as a hypothesis and not as a finding. What the data do show is the time scale: within the campaign itself the scene was static, with 0.0 px displacement measured between the first and the last clip of the night, so the pose changes between deployment sessions rather than within them.

The operational consequence was quantified directly by re-projecting the same recorded footpoints through both the pre-displacement and the re-fitted homography. Because detection and tracking are identical in the two cases, the difference is attributable to the calibration alone. Using the stale calibration, the mean absolute error at the enforcement line rose from 3.19 km/h to 7.74 km/h (a factor of 2.42) and the bias from −2.60 km/h to −7.38 km/h. In 3 of the 39 evaluable passes the stale calibration changed the enforcement outcome: vehicles travelling at 40–43 km/h by GNSS were read at 35–37 km/h with the correct calibration (above the 35 km/h tolerance of DS 011-2026-MTC) but at 28–29 km/h with the stale one (below it). The direction of the error matters legally: an undetected camera shift of the kind observed here produces false negatives—violations that go undetected—rather than unwarranted penalties.

We therefore recommend that homography-based enforcement deployments verify calibration periodically, at minimum after any reinstallation and at intervals consistent with local weather exposure. We note that the ORB-based automatic displacement check we implemented proved unreliable when comparing frames captured under different illumination—a daytime reference against a nighttime frame produced a spurious 267 px displacement—so verification should compare frames acquired under comparable lighting, or re-measure the control points directly.

### 3.9. License Plate Recognition

#### 3.9.1. Detection Stage

The plate recognition pipeline operates on the frontal camera stream processed by Jetson .28. The detection stage employs the YOLO26n_suelo model (Section 3.4), which simultaneously detects vehicles (seven classes: A, M, MB, PU, MT, CP, CG) and plate regions (“plate” class) in a single inference pass. This joint detection eliminates the need for a separate vehicle-plate association step, since ByteTrack maintains persistent tracking identities across frames and each detected plate is spatially associated with its enclosing vehicle bounding box through geometric containment.

#### 3.9.2. CCT Character Recognition

Detected plate regions are cropped from the frame and passed to a pre-trained Compact Convolutional Transformer (CCT) [77] model (cct-s-v2-global-model, 5 MB, ONNX format), obtained from the open-source fast-plate-ocr library [78], for optical character recognition. The CCT model operates on CPU (device=’cpu’), a deliberate architectural choice that avoids GPU contention with the YOLO detection model and leverages the available CPU cores of the Jetson Orin NX during inference cycles that occupy the GPU. Each plate inference requires approximately 43.1 ms on CPU, which is sufficient for the target throughput given that OCR is triggered every 3 frames per tracked plate rather than at every frame.

The CCT model was selected after a systematic comparison against EasyOCR, in which 35 plate images from the deployment site were evaluated. CCT achieved correct full-plate readings in all 35 images (35/35), while EasyOCR produced no correct full-plate readings (0/35), failing primarily in the character segmentation of the Peruvian format. This decisive advantage justified the adoption of CCT, which reads the entire cropped plate region directly in a single forward pass, without requiring separate per-character segmentation prior to recognition.

#### 3.9.3. Temporal Aggregation and Format Validation

To reduce character-level OCR errors, the system implements a temporal aggregation strategy through ByteTrack persistent tracking. Each tracked vehicle is subjected to periodic OCR re-reading (every 3 frames while the plate region remains detected) and multiple readings per vehicle are consolidated. Peruvian plates follow a six-character alphanumeric format; a regular-expression-based validator filters readings that do not conform to this structure, discarding partial or spurious detections.

Known OCR confusion pairs were identified during field testing: A↔V (similar diagonal strokes), L↔1 (vertical stroke ambiguity), and R↔P (open versus closed counter). These confusions are documented as a system limitation; a Levenshtein-distance-based post-processor for duplicate plate consolidation is proposed as future work (Section 6).

### 3.10. Traffic Violation Classification

#### 3.10.1. Speed Violation Classification (Code M20)

The system classifies speed violations in accordance with Peruvian National Traffic Regulation (DS 025-2021-MTC) as modified by DS 011-2026-MTC. The speed limit established for *jirones* (narrow urban streets) is 30 km/h. The recently introduced camera-based enforcement tolerance (DS 011-2026-MTC, Art. 168-A.1) establishes a 5 km/h threshold below which camera-detected excesses are not sanctioned. The system applies this tolerance by triggering violation classification only when the measured speed exceeds 35 km/h (30 + 5 km/h tolerance).

Three severity levels are defined under violation code M20, where the excess is computed as $\Delta v=v_{\mathrm{measured}}-30$ km/h, as listed in Table 10:

#### 3.10.2. Traffic Signal Violation Detection (Code M30)

The traffic signal violation detection module implements temporal correlation with the fixed-cycle traffic light located at the downstream intersection of Jr. Carlos F. Vivanco. The traffic light operates with a 98-s fixed cycle: green phase (50 s), amber phase (3 s), and red phase (45 s). A reference calibration timestamp (17:57:06 on 6 July 2026) was established through direct observation and verified against the real-time signal state during field testing.

The current signal state at any time *t* is computed via modular arithmetic:

(6) $$\phi \left(t\right)=\left(t-t_{\mathrm{ref}}\right)\;\mathrm{mod}\;98$$

where $t_{\mathrm{ref}}$ is the calibration timestamp in seconds, and phase ϕ maps to green (0≤ϕ<50), amber (50≤ϕ<53), or red (53≤ϕ<98). The detection line for red-light violations is positioned at L2 (pixel row y=665), approximately 6 m before the physical stop line, providing a conservative detection margin.

To prevent false positives from vehicles stopping near but not at the detection line (for example, vehicles decelerating in queue), a minimum speed filter of 15 km/h is applied: only vehicles crossing L2 during a red phase at speeds above 15 km/h are flagged as M30 violations. Red-light violations carry a fine of 12% UIT (S/ 660) and 50 demerit points.

An important observation is that the traffic signal module operates as an independent calibrated component. The module code was installed and successfully tested on Jetson .27, confirming that the modular arithmetic approach correctly tracks the fixed traffic light cycle over extended operating periods. However, its integration into the real-time streaming pipeline is under development at the time of writing this article and constitutes planned future work.

#### 3.10.3. System Limitations and Regulatory Status

The system operates as a prototype for presumptive violation detection and does not constitute a certified cinemometer under the Peruvian metrological standards (NMP 013:2010). Certified speed enforcement in Peru requires radar- or laser-based cinemometers with periodic calibration by INACAL (National Institute of Quality). SUTRAN operates only 14 certified cinemometers across the entire national territory, concentrated on national highways with high accident rates, evidencing the inaccessibility of this technology for municipal governments with restricted budgets. The present system is designed as a complementary tool for municipal traffic monitoring, capable of generating preliminary violation records that would require confirmation with certified instruments before formal sanction. This regulatory positioning is consistent with the system deployment in the municipal Serenazgo booth, where operators use it as a real-time awareness tool rather than as a primary enforcement device.

## 4. Results

### 4.1. Homography Calibration Validation

The homography matrix H computed from the six calibration points using cv2.findHomography with the ordinary least-squares method (method=0) produced the following 3×3 projective transformation:

(7) $$H=\left[\begin{array}{ccc} 1.94956194\times 10^{-2} & 1.73303119\times 10^{-1} & -1.19532490\times 10^{2}\\ 1.03047597\times 10^{-1} & 1.32529000\times 10^{-2} & -3.47915229\times 10^{1}\\ 8.31626176\times 10^{-5} & 8.23428622\times 10^{-3} & 1.00000000 \end{array}\right]$$

The per-point reprojection error, computed as the Euclidean distance between the transformed pixel coordinates and the corresponding ground-truth metric coordinates, is presented in Table 11. The longitudinal axis is defined as the unit vector from the near baseline (midpoint of P1–P2) to the far baseline (midpoint of P3–P4); the longitudinal error of each point is the projection of its Euclidean error vector onto this axis, and the lateral error is the orthogonal component. The mean reprojection error is 18.3 cm, with a maximum individual point error of 29.7 cm at P2 (right curb, near zone). The longitudinal component of the error is 4.4 cm on average, which is the metrically relevant quantity for speed estimation since only longitudinal displacement contributes to the vehicle velocity vector; the remainder of the per-point error is predominantly lateral (e.g., P2 and P4), which does not bias the speed estimate to the same degree.

Applying H to the pixel positions of L1 (y=94) and L2 (y=665) yields a metric distance of approximately 52.5 m, substantially shorter than the 84.1 m orthophoto-measured L1–L2 separation. This discrepancy does not indicate an error in H; rather, it reflects that L1 lies beyond the calibrated span of the homography (the six control points cover approximately 39.4 m longitudinally, from the near baseline P1–P2 to the far baseline P3–P4 at pixel row y=142), so a direct comparison between the two figures is not methodologically meaningful. Accordingly, the reliable operating zone of the homography-based speed estimation is bounded to 0–42 m from the near baseline, and the 84.1 m figure is reserved exclusively for the independent line-crossing method, which measures transit time directly between the two physical reference lines and is therefore unaffected by this limitation.

A seventh candidate point (P7), initially considered during calibration, was discarded due to an anomalous reprojection error of 184 cm. Post-hoc analysis revealed that P7 was located on a slightly elevated pavement patch, violating the planar surface assumption of the homography. This finding is documented as a methodological note: the ground-plane assumption underlying planar homography is sensitive to real elevation deviations from the calibrated plane, not to sub-centimeter irregularities—an elevation difference of approximately 0.5 m at the measurement point (with camera height H≈9 m and horizontal distance d≈30 m, the displacement h·d/H≈0.5×30/9≈1.67 m, consistent with the observed 1.84 m reprojection error) is required to produce the observed error, and calibration point selection should prioritize features clearly on the dominant road surface plane.

### 4.2. Detection Model Comparison

The comprehensive comparison of models M1–M6 on their respective test sets (Table 6) reveals four key trends. First, the transition from YOLOv8 (M1) to YOLO11 (M2) produced marginal improvements (Box mAP50: 0.859 → 0.863, +0.4 pp), consistent with the incremental nature of architectural refinements in this generation. Second, the transition to YOLO26 (M3) produced substantial gains (Box mAP50: 0.903, +4.0 pp over M2), reflecting the impact of the YOLO26 architectural innovations (NMS-free inference, removal of Distribution Focal Loss, ProgLoss with Small-Target-Aware Label Assignment, and the MuSGD optimizer). Third, the progressive three-phase training of M4 produced the highest metrics of the segmentation series (Box mAP50: 0.920, Mask mAP50: 0.891), validating the progressive resolution strategy for scenarios with high aspect-ratio variation in object sizes. Fourth, the transition from segmentation to detection (M4 → M5) sacrificed 6.4 percentage points in Box mAP50 (0.920 → 0.856) in exchange for a 4× inference acceleration (5 FPS → 20.2 FPS on Jetson Orin NX), a trade-off justified by the operational requirements of real-time monitoring.

#### Per-Class Performance of M5

The per-class analysis of M5 (Table 7) reveals that the mototaxi class (MT) achieves the highest mAP50 (0.949) despite being a minority class before augmentation (1286 instances, 2.9% of the original dataset). This result demonstrates the effectiveness of the ground-aware copy-paste augmentation strategy for region-specific vehicle types. The rural combi class (RC) exhibits the lowest performance (mAP50 of 0.695), attributable to two factors: visual similarity with automobiles (confusion matrix analysis reveals that 31% of RC instances are misclassified as A) and a limited number of original training instances (359 instances, 0.8% of the original dataset). The consistency between validation and test metrics (Box mAP50: 0.856 in both sets) confirms the absence of overfitting.

Figure 7 presents the normalized confusion matrix underlying this analysis, computed on the held-out M5 test set.

### 4.3. Speed Estimation Accuracy

#### 4.3.1. Preliminary and Extended Nocturnal Campaigns (Superseded)

Two earlier field campaigns preceded the corrected-pipeline validation reported below; both are superseded by it and are reported in full, including their validation tables and figures, in Supplementary Material File S1. A first 3-point GPS validation (6 July 2026, VelocX speedometer app) gave MAE = 0.33 km/h over a 0–14 km/h range, but the sample (N=3) was too small to characterize system accuracy and motivated a larger campaign.

A subsequent 24-pass nocturnal campaign (15 August 2026, dashboard speedometer and GNSS Doppler references) attempted this broader validation but was confounded by two previously unreported deployment failure modes, both diagnosed during this work. First, from pass 7 onward the aerial camera’s RTSP sub-stream silently renegotiated from the calibrated 1280 × 720 resolution to 2304 × 1296, a 1.8× scale change that the deployed script did not detect before computing the homography; this produced a systematic underestimation (peak-speed ratio 0.548, consistent with the 1/1.8=0.556 geometric prediction) in 18 of the 24 passes, leaving only two passes (Group A, N=2) free of the confound—insufficient to characterize accuracy statistically. Second, an independent display-path defect caused the on-screen speed to hold its last value instead of decaying to zero when a tracked vehicle stopped, in 8 of the 18 affected passes; the September campaign below later confirmed this defect was unrelated to the resolution issue and never affected the underlying measurement chain or violation decisions. A mandatory resolution check was implemented and deployed as mitigation for the first defect.

#### 4.3.2. Corrected-Pipeline Validation Campaign (18–19 September 2026)

The mitigation described in Section 4.3 was re-validated in a dedicated nocturnal campaign on 18–19 September 2026 (23:00–00:32 local time) comprising 40 controlled passes of an instrumented vehicle. GNSS Doppler velocity recorded at 1 Hz with a BasicAirData GPS Logger served as the reference. Doppler-derived speed from a consumer GNSS receiver is specified to about 0.1–0.5 km/h, which is roughly an order of magnitude finer than the errors being characterised; unlike the August campaign, no dashboard speedometer was recorded, so a single reference was used throughout.

The campaign covered 16.9–48.8 km/h (per-pass GNSS maximum), spanning both the 30 km/h statutory limit on Jr. Carlos F. Vivanco and the 35 km/h camera tolerance of DS 011-2026-MTC. The upper bound was set by road safety: the site is a congested urban corridor and higher speeds could not be attempted responsibly. The range therefore covers the enforcement-relevant band that earlier campaigns did not reach, but does not extend to free-flow highway speeds.

##### Vehicle Attribution

Each pass was recorded with the resolution check enforced and reprocessed offline. Attribution of the test vehicle to a specific track was performed by an operator reviewing the annotated video for each pass, not by an automatic criterion. This is deliberate: selecting the track whose speed best matches the reference would be circular, and selecting by position alone proved unreliable in practice—in one pass (32) the automatic position-based criterion returned no candidate at all, while the operator identified the vehicle without difficulty. All 40 passes were attributed this way, and the confirmed track identifiers are provided in the Supplementary Material.

##### Statistical Unit

The unit of analysis is the *pass* (N=40), not the individual paired reading. Readings within a pass are strongly autocorrelated—the same vehicle, the same filter state, video at 20 fps against a 1 Hz reference—so treating the 304 paired readings as independent observations would substantially overstate precision. Confidence intervals were obtained by bootstrap resampling of passes. The primary metric is the single reading taken as the vehicle crosses the enforcement line at s=10 m, which is both the operationally meaningful quantity for a speed-enforcement camera and independent across passes; 39 of the 40 passes yielded a valid reading at that line. MAPE is deliberately not reported: passes include signal-controlled stops, and division by a near-zero reference makes the statistic unstable and uninformative.

##### Field Validation of the Homography

Because the GNSS logger measures position as well as speed, the campaign also provides an independent field check of the calibration that does not depend on the speed estimator. After temporal alignment, the longitudinal position reported by the system agreed with the GNSS track to within a bias of +1.14 m (95% CI −0.03 to +2.33 m), MAE 3.96 m and RMSE 4.92 m across the 40 passes over a 41 m measurement zone. This residual is at the noise floor of consumer GNSS horizontal positioning (2–5 m) and an order of magnitude larger than the 31.1 cm control-point reprojection error, which is the expected ordering if the calibration is sound. This is a considerably stronger check than reprojection error at the control points alone, since it is evaluated along the whole trajectory against an independent instrument.

##### Speed Accuracy of the Deployed Estimator

At the enforcement line the deployed constant-velocity (CV) Kalman filter achieved MAE 3.19 km/h (95% CI 2.59–3.85), RMSE 3.64 km/h and bias −2.60 km/h (95% CI −3.27 to −1.44) over N=39 passes, with Bland–Altman limits of agreement of [−7.7,+2.5] km/h, a Deming slope of 0.825 and Lin’s concordance correlation coefficient of 0.915 (Figure 8 and Figure 9). The underestimation is systematic and the Deming slope departs clearly from unity, which motivated the diagnostic reported next.

##### Mechanism of the Systematic Underestimation

The error is not a start-up transient. Initialising the filter velocity from the first two position measurements, rather than from zero, left the results unchanged to three significant figures in the 39 of 40 passes where the initialisation branch was exercised. Instead, the error is the steady-state lag of a constant-velocity model tracking an accelerating target. Across passes, the speed error correlates with the mean vehicle acceleration within the zone at r=−0.727, with a slope of −4.61 km/h per m s^−2^ (Figure 10), corresponding to an effective filter time constant of about 1.28 s. Grouping passes into acceleration terciles, the mean error grows from −0.66 km/h at +0.11 m s^−2^ to −4.72 km/h at +1.00 m s^−2^.

##### Range-Dependent Accuracy

Speed error grows with distance from the camera, from a mean absolute error of 2.08 km/h for s<10 m to 9.49 km/h for s≥30 m (Figure 11). The same trend is present in the unfiltered position derivative, which identifies it as a property of the projective geometry—at range, a pixel subtends a much larger ground distance—rather than of the filter. This supports siting the enforcement line in the near field, as is done here.

##### Mechanism of the Systematic Underestimation (Continued)

This mechanism is aggravated by the geometry of the application. Fitting |error|(t)=aexp(−t/τ)+c to the readings gives τ=4.33 s (95% CI 2.12–8.38 s), while the transit time across the 41 m zone is 4.9 s at 30 km/h, 3.7 s at 40 km/h and 3.0 s at 50 km/h. At enforcement-relevant speeds the vehicle therefore leaves the measurement zone after less than one filter time constant, and the estimate never settles. Figure 12 shows this directly for the pass with the highest mean acceleration.

##### Proposed Correction and Its Validation

Because the residual is driven by unmodelled deterministic acceleration, the principled remedy is to extend the motion model rather than to inflate the process noise. We evaluated a constant-acceleration (CA) model with state $\left[x,y,v_{x},v_{y},a_{x},a_{y}\right]$ and white-noise jerk, retaining every other element of the pipeline unchanged. With $\sigma_{\mathrm{jerk}}=1.0$ m s^−3^—a value consistent with vehicle dynamics, and an interior optimum of the search grid—the MAE at the enforcement line falls to 1.26 km/h, RMSE 1.79 km/h, bias −0.43 km/h, Deming slope 1.009 and CCC 0.982, with limits of agreement of [−3.9,+3.0] km/h. Crucially, the dependence on acceleration is eliminated: the correlation falls from r=−0.727 to r=+0.003 and the slope from −4.61 to +0.01 km/h per m s^−2^. That the predicted mechanism disappears when the corresponding term is added to the model is stronger evidence than the improvement in MAE alone.

To ensure the tuning parameter is not fitted to the evaluation data, all comparisons were repeated under leave-one-pass-out cross-validation, in which the parameter is selected without reference to the pass being evaluated. The cross-validated figures are MAE 1.32 km/h for the CA model, 1.36 km/h for a causal backward-window position derivative and 1.40 km/h for the CV model with inflated process noise, against 3.19 km/h for the deployed configuration (Table 12). The CV model with inflated noise requires $\sigma_{a}\approx 60$ m s^−2^ (≈6 g) to approach this performance, which has no physical interpretation and illustrates that the process noise is being used to absorb a deterministic term. All estimators compared here are strictly causal. A centred-window derivative, which uses future frames and is therefore not deployable, attains MAE 1.33 km/h and is reported only as an upper bound on what the position signal supports.

##### Behaviour at Stops

The August campaign left open whether the system correctly reports zero speed for vehicles stopped at the signal. Across the paired readings in which the GNSS reference was below 2 km/h, the reported speed averaged 0.61 km/h (median 0.23, maximum 3.07), with 86% of readings below 2 km/h and 100% below 5 km/h. Stops are therefore detected correctly by the measurement chain.

The apparent failure observed in August was traced to the display path rather than to the estimator. When a track leaves the measurement zone (s<0), the update routine resets the filter and returns early, but the returned dictionary carries the last stored speed because the reset does not clear it, and the flag indicating a valid measurement is set to false. The violation detector and the CSV log both test that flag; the on-screen annotation did not. Vehicles that stopped just beyond the zone exit therefore retained their last displayed value indefinitely—a frozen reading of 16.49 km/h persisting for more than 8 s is visible in pass 5 of the present campaign—while the logged measurements and the enforcement decisions were unaffected. This reproduces at the correct 1280 × 720 resolution, which means the resolution mismatch was not its cause; the hypothesis advanced in Section 4.3 is accordingly withdrawn. The defect is one of visualisation and has been corrected by testing the validity flag before rendering.

##### Vehicle Classification as a Function of Range

The same range-dependent projective geometry discussed above also affects classification. The test vehicle, a pickup (class PU) throughout, was classified correctly in only 6 of 40 passes under a whole-track majority vote, because it is labelled as an automobile while distant: 99.0% of frames with a bounding-box height below 30 px were labelled A, against 96.5% labelled PU above 120 px, with the crossover near 60–80 px (s≈18 m). Mean detection confidence was also lower for the incorrect label (0.787 versus 0.891). Restricting the majority vote to the near field (s<20 m, or box height above 80 px) recovers the correct class in all 40 passes. Since plate reading and violation classification occur in the near field in any case, this restriction is operationally free, and we adopt it.

##### Equivalence Between Offline Reprocessing and the Deployed Edge Hardware

The validation above was obtained by reprocessing the recorded passes on a workstation using the PyTorch FP32 checkpoint and every frame of the recording, whereas the deployed node runs a TensorRT FP16 engine and sustains 20.0–22.3 FPS against a 25 fps camera. Both differences act on the measurement chain: quantisation shifts detection confidences and can drop marginal detections, which fragments tracks and reinitialises the filter, while processing fewer frames changes the filter update interval. Reporting the workstation figures as characterising the deployed system therefore requires evidence, not assertion.

Ten of the forty passes, selected to span the speed range (17.1–48.8 km/h) and to include three signal-controlled stops, were reprocessed on the deployment node itself under two conditions: all frames, which isolates the effect of FP16 quantisation; and uniformly decimated to 80% coverage, which reproduces the frame utilisation of the live deployment. Detection, tracking, homography and filter code were otherwise identical, and the recalibrated homography was installed as a separate module so that the production configuration was left untouched. Because ByteTrack assigns identifiers independently in each run, the test vehicle was re-identified in every output by matching the s(t) trajectory against the operator-confirmed track, with trajectory matching residuals of 0.04–0.10 m.

FP16 inference produced 2.1% fewer detections than FP32 (23,395 versus 23,896), and decimation to deployment frame rate reduced the total by 21.8%. Neither materially affected the measured speed. The paired difference in the reading at the enforcement line between the edge node and the workstation was −0.02 km/h on average (SD 0.08, maximum absolute difference 0.17 km/h), and the mean absolute error over these ten passes was 0.64 km/h on both platforms, rising only to 0.68 km/h at deployment frame rate (Table 13). The detections lost to quantisation and decimation are therefore marginal ones—low-confidence boxes on distant vehicles—rather than detections of the tracked vehicle.

We note that the mean absolute error over this ten-pass subset (0.64 km/h) is lower than over the full campaign (1.26 km/h); the subset was chosen to span the operating envelope for a platform comparison, not to estimate accuracy, and the quantity of interest here is the difference between platforms rather than its absolute level. The conclusion is that the offline results can be attributed to the deployed hardware, and that the constant-acceleration filter retains its advantage on the edge device.

### 4.4. License Plate Recognition Performance

#### 4.4.1. M6 Versus YOLO26n_suelo Comparison

Table 14 presents the comparison between the two candidate models for the plate detection stage: M6 (trained exclusively on the 1513-image proprietary dataset) and YOLO26n_suelo (trained on the 7555-image combined dataset with three additional Roboflow sources). Both models were evaluated on the same held-out proprietary test set (150 images) to enable direct comparison. The plate-class mAP50 is 0.815 for M6 and 0.807 for YOLO26n_suelo, indicating equivalent performance for Peruvian plate detection.

This negative result is reported transparently as a methodological finding. The initial expectation was that the addition of external plate data (∼6922 international plate instances from three Roboflow datasets) would improve the plate detection performance through the increased training volume. Post-hoc analysis suggests two factors explaining the observed equivalence. First, the domain gap between international plate formats (varying character counts, fonts, aspect ratios, and background colors) and the standardized Peruvian format (six alphanumeric characters, specific font, white background with black characters) is larger than the initial hypothesis assumed. Second, the proprietary 1513-image dataset already captures the relevant intra-domain variability (illumination, angle, distance, occlusion) sufficiently to saturate the learning capacity of the YOLO26n architecture for this specific task.

For deployment, the YOLO26n_suelo model was retained as the operational plate detector, based on the marginal superiority in overall mAP50 (0.851 vs. 0.845) and its slightly better performance on the vehicle classes (which share detection responsibilities with the plate class in the multi-task pipeline). The finding is nonetheless documented as guidance for future ALPR deployments in developing regions: local data collection with domain-specific formats provides comparable results to hybrid strategies combining local and international data.

#### 4.4.2. OCR Performance: CCT Versus EasyOCR

The systematic comparison between CCT and EasyOCR on 35 plate images extracted from the deployment site yielded a decisive result: CCT achieved 35/35 correct full-plate readings (100% accuracy), while EasyOCR achieved 0/35 correct full-plate readings (0% accuracy). Analysis of the EasyOCR failure modes revealed systematic character segmentation errors on the Peruvian format, in which EasyOCR frequently merged consecutive characters or split single characters into multiple detections. The CCT model, in contrast, produced consistent character-level segmentation and recognition across all evaluated plates.

This 100%-versus-0% result cannot be extrapolated as a general benchmark, since the sample size is limited and the plates were captured under favorable conditions (frontal angle, adequate illumination). Nevertheless, it justifies the operational choice of CCT for the deployed system.

#### 4.4.3. Field-Validated Plates

During the nighttime field testing on 6 July 2026, the operational system correctly recognized 14 Peruvian license plates from vehicles passing through the monitored corridor. Table 15 lists the recognized plates and their corresponding vehicle types.

The recognized plates span the full range of vehicle types observed at the site: automobiles (predominant), microbuses, motorcycles, and pickup trucks. Character-level analysis of the recognized plates reveals the practical occurrence of the known OCR confusion pairs identified in Section 3.9.3: the character sequences “LE4145” (containing the L-1 pair) and “BBC584” (containing repeated B characters that could be confused with the number 8) were recognized correctly through the temporal aggregation strategy, which consolidates multiple readings per tracked vehicle.

Figure 13 shows representative examples of plate recognition on the operational system, including the tracking overlay, plate localization, and OCR output panel.

### 4.5. Real-Time System Performance

Table 16 presents the sustained runtime performance of both processing nodes measured during continuous 4-h operation on 6 July 2026 (17:00–21:00). Both subsystems sustain throughput above the 20 FPS threshold considered acceptable for real-time traffic monitoring, without stream interruptions or system instability during the evaluation window. The processing rate of 20.0–22.3 FPS from a 25 FPS source corresponds to a frame utilization rate of 80–89%, with non-processed frames discarded by design rather than lost to system failure.

The GPU utilization difference between the two nodes (78–85% on Jetson .27 versus 65–72% on Jetson .28) reflects the complementary strategy of running CCT on CPU (Jetson .28) versus the fully GPU-bound speed pipeline (Jetson .27). The 4-h continuous operation without stream interruptions confirms the thermal stability of the actively fan-cooled Jetson Orin NX enclosures under the observed ambient temperature conditions (18–22 °C indoor). The plate node’s sustained FPS range (29–36) exceeds its 30 FPS camera source rate at its upper bound; this reflects loop-iteration throughput of the detection and tracking pipeline rather than a strict count of newly captured camera frames, since the OCR stage is triggered only every 3 frames per tracked plate (Section 3.5) while detection and tracking run on every loop iteration. Reported FPS values should therefore be interpreted conservatively, as processing throughput of the pipeline rather than an exact camera frame-capture rate.

Figure 14 presents the integrated operational view of the complete system, showing both processing dashboards simultaneously on the Serenazgo display.

### 4.6. Daytime Versus Nighttime Operation

Field testing was conducted under both daytime (14:00–17:00) and nighttime (19:00–21:00) conditions to characterize the system performance across the operational envelope. The four metrics reported in Table 17 are defined as follows. Vehicle detection rate is the proportion of ground-truth vehicles that were correctly detected by the YOLO26n model (confidence ≥0.6) in at least one frame during their transit through the camera field of view. Tracking persistence is the average number of consecutive frames for which a ByteTrack identifier remained continuously associated with a given vehicle before an identity switch or track loss occurred. Plate reading success is the proportion of vehicles with a legible plate, as judged by manual review, for which the temporal-consensus OCR output exactly matched the ground-truth plate text. False positives is the count of spurious vehicle detections (confirmed non-vehicle or duplicate detections) that persisted long enough to pass the ByteTrack minimum-persistence filter, normalized per hour of operation. Ground truth was established by the first author through manual frame-by-frame review of 200 consecutive vehicles per condition from the recorded video streams.

Table 17 summarizes the observed differences, including 95% Wilson score confidence intervals for the two proportion-based metrics (detection rate and plate reading success); the interval is not applicable to the two rate/average-based metrics (tracking persistence, false positives).

The observed nighttime performance degradation of 4.5 percentage points in detection rate is attributable primarily to contour loss in dark-colored vehicles under low-illumination conditions. The colonial architecture of Ayacucho contributes additional challenges: the yellow sodium street lighting produces color shifts that affect the YOLO detector’s confidence for certain vehicle classes, and moving shadows from vehicle headlights on adjacent colonial mansion facades occasionally trigger false detections that are subsequently filtered by the ByteTrack minimum-persistence threshold.

Figure 15 illustrates the operational speed estimation under three representative conditions: daytime with clear visibility, nighttime under street lighting, and daytime with strong sun halo interference on the camera lens.

## 5. Discussion

### 5.1. Positioning Against State-of-the-Art

The obtained results position the proposed system within the range of the recent literature on vision-based speed estimation. Over the 40-pass campaign of Section 4.3.2, the configuration deployed during the campaign attained MAE 3.19 km/h at the enforcement line and the proposed constant-acceleration filter 1.32 km/h under cross-validation, against MAE 1.694 km/h reported by Rodríguez-Rangel et al. (YOLOv3 + Kalman filtering) [10], 1.34 km/h by Kesli and Kilicarslan (Speed-YOLOv8) [35], 3.38 km/h by Sangsuwan and Ekpanyapong (laser reference) [32] and 3.2 km/h by Islam et al. (ByteTrack) [31]. Three qualifications apply. First, the statistical unit here is the pass rather than the individual reading, and N=40 remains smaller than the N>100 samples of several of those studies. Second, the validated band (16.9–48.8 km/h) covers the enforcement-relevant range around the 30 km/h limit and the 35 km/h tolerance, but not the 20–80 km/h spans typically reported elsewhere, and all passes used a single vehicle at a single site under nocturnal conditions. Third, the comparison is between figures obtained under different references and protocols, and headline MAE values across such studies are not strictly commensurable. Two earlier and methodologically weaker estimates (MAE 0.33 km/h, N=3, 0–14 km/h; and a Group A peak-to-peak error of −4.1 km/h, N=2, 16–22 km/h, both in Section 4.3) are retained for the record but are superseded by the present campaign and should not be read as characterizations of system accuracy; the peak-to-peak protocol in particular carries a known directional bias, since the peak of a noisy reference trace is biased upward and that of a smoothed estimate downward.

The sustained real-time throughput of 20.0–22.3 FPS for speed estimation and 29–36 FPS for plate recognition, achieved on Jetson Orin NX with TensorRT FP16 optimization, exceeds the performance reported by Ammar et al. (17.1 FPS on Jetson Xavier AGX) [20], the most direct comparable system in the literature. The key architectural difference lies in the distributed two-node approach adopted here, which eliminates the GPU contention observed when running both detection models on the same device. This design decision, empirically validated during preliminary deployment, is a transferable finding for edge computing deployments in resource-constrained scenarios.

The dual-method architecture (homography plus line crossing) is proposed as an original combination not previously reported in the literature. Existing systems adopt only one of the two paradigms, inheriting their respective limitations: line-crossing methods fail during vehicle stops [7,8], while homography-based methods are more sensitive to detection noise at long distances [10,11]. The architectural intent is for the complementary integration of both methods to allow continuous operation in congested traffic conditions (where line crossing would fail) with cross-validation available when vehicles complete the segment traversal; however, this cross-validation capability has not yet been quantitatively demonstrated. In the field session logged for direct comparison (Table 18, n=26 tracked vehicles), no vehicle completed a full L1–L2 crossing, so no paired homography/line-crossing measurement for the same vehicle is currently available; congestion and the traffic-light cycle at the downstream intersection meant most tracked vehicles stopped before reaching L2, which is itself consistent with the motivation for the homography-based method described above, but leaves the quantitative same-vehicle cross-validation of the two methods as future work, pending a field session focused on free-flowing traffic.

### 5.2. Local Versus External Data

The negative result obtained in the plate detection comparison (M6 with local data 0.815 vs. YOLO26n_suelo with combined data 0.807) constitutes a methodological finding relevant to the ALPR literature in developing regions. The initial hypothesis, based on the general principle that larger training volumes improve model performance, was that combining the local 1513-image dataset with three additional Roboflow datasets (∼6922 international plate instances) would yield superior plate detection performance. The observed equivalence, verified on the same test set, indicates that the domain gap between international plate formats and the standardized Peruvian format outweighs the benefit of the additional training volume.

This finding aligns with the recent discussion by Xu et al. on cross-regional generalization as a persistent open problem in ALPR [19] and provides quantitative evidence supporting the “local data first” principle for the deployment of ALPR systems in regions with standardized national plate formats. For ALPR deployments in developing countries, the practical implication is that limited local data collection can be more effective than hybrid strategies combining international sources, contradicting the common assumption in the field that additional data always improves performance.

### 5.3. Limitations

Eleven limitations of the present work are transparently documented to inform the interpretation of the results and to guide future improvements.

The first limitation concerns the scope of the speed-accuracy characterization. The 40-pass campaign of Section 4.3.2 supersedes the two earlier and much weaker estimates (N=3, 0–14 km/h, 6 July 2026; and N=2, 16–22 km/h, 15 August 2026), and establishes accuracy over 16.9–48.8 km/h against a GNSS Doppler reference. Its scope is nonetheless bounded in four respects. All 40 passes used a single instrumented vehicle, so speed accuracy—and likewise the range-dependent classification behaviour reported in the same section—is established for one vehicle type at one site. The validated band is bounded above at 48.8 km/h because the corridor is congested and higher speeds could not be attempted safely, so performance at free-flow highway speeds is not established. A single reference instrument was used, so no cross-check between independent references is available, although GNSS Doppler speed is specified an order of magnitude finer than the errors measured. Finally, vehicle attribution was performed by a single operator; an independent second reading on a subset of passes would allow inter-observer agreement to be reported.

Speed validation was moreover conducted exclusively at night. This is a constraint of the site rather than a choice of convenience: Jr. Carlos F. Vivanco is one of the busiest corridors in the historic center, and during daytime it is congested to the point that a test vehicle can neither reach nor sustain a steady speed through the measurement zone—an instrumented pass under daytime conditions would be limited to approximately 20–25 km/h by traffic alone, well below the enforcement-relevant band this work targets, and attempting it would additionally risk a collision in dense traffic. For the same reason, extending the campaign to additional vehicle types (e.g., motorcycles or buses) was not attempted: the homography-based estimator measures the metric trajectory of a single tracked footpoint and does not otherwise condition on vehicle class, so the characterized error sources—the constant-velocity motion-model lag under acceleration and far-zone footpoint noise (Section 5.3)—are properties of the tracking and filtering pipeline rather than of a particular vehicle’s dynamics. The component expected to vary across vehicle types is detection and footpoint-extraction reliability, which is characterized separately, per class, by the M5 confusion matrix (Figure 7) rather than repeated here for each class against GNSS. Daytime operation of the system is nonetheless characterised in Table 17, which reports detection and tracking rates for both conditions on 200-vehicle samples; what the present campaign does not establish is daytime *speed* accuracy. We note that the nocturnal condition is the more demanding one for detection, so the reported speed accuracy is unlikely to be optimistic on that account. Finally, the constant-acceleration filter proposed in Section 4.3.2 was validated on recorded video, reprocessed both on a workstation and on the deployment node (Section Equivalence Between Offline Reprocessing and the Deployed Edge Hardware); it has not yet been exercised against a live RTSP stream in continuous operation, which remains the final deployment step.

The second limitation is the reliance on a smartphone camera as the frontal video source. Although the IP Webcam application provides sufficient resolution and frame rate for the current implementation, the WiFi transmission introduces variable latency (0.75–50 ms depending on channel conditions) and depends on smartphone battery availability. Replacement with a wired IP camera is recommended for permanent deployment.

The third limitation is the nighttime degradation in vehicle class classification. The reported 4.5 percentage-point decrease in detection rate under nighttime conditions primarily affects dark-colored vehicles under yellow sodium lighting. Complementary training with a curated nighttime image set (approximately 1300 currently unlabeled nighttime images are available from the deployment site) is expected to reduce this gap.

The fourth limitation is the operational validation at a single intersection. Although the calibration procedure is designed to be transferable to any new location through the drone-orthophoto pipeline, the system has not been validated at a second site. Deployment at Puente Nuevo (an adjacent site with different geometric and traffic characteristics) is planned to evaluate the transferability of the approach.

The fifth limitation concerns OCR confusions between visually similar characters (A↔V, L↔1, R↔P). Although the temporal aggregation strategy mitigates these confusions through multiple readings per tracked vehicle, occasional errors persist. A Levenshtein-distance-based post-processor for duplicate plate consolidation is proposed as future work.

The sixth limitation is the current absence of correlation between the speed and plate subsystems. The two nodes operate independently, and the vehicles measured by the speed subsystem are not currently linked to the plate readings from the plate subsystem; the demonstrated functionality of each node (continuous speed estimation on one hand, real-time plate reading on the other) should therefore not be conflated with the generation of a complete, per-vehicle violation record, which requires fusing the two. Because both cameras observe the same street segment from the same installation point, a candidate matching strategy is temporal correlation: a vehicle detected by the aerial node should appear at the frontal node within a time window predictable from its measured speed and the known distance between the two camera fields of view, with vehicle-class consistency (both subsystems already classify detections into the same taxonomy) as a secondary filter, in the spirit of signature-matching approaches used for cross-sensor vehicle reidentification in road networks [79]. This approach is expected to be ambiguous when multiple vehicles of the same class travel through the corridor within the same time window, a common occurrence under the congested conditions this system targets. Reducing that ambiguity would likely require finer spatial discrimination than temporal correlation alone can provide, for example lane-level separation combined with homography-based distance estimation between the two vantage points. The present deployment mounts the aerial and frontal cameras on an existing building (the Serenazgo booth), which is not guaranteed to be available at other candidate sites; a centrally mounted gantry or pole-arm spanning the roadway, a configuration standard in commercial ANPR deployments, would avoid this dependency and provide a consistent vantage point over both traffic directions. At the conceptual level, such a structure would consist of a single roadside pole supporting a horizontal arm clearing the carriageway at a height sufficient to remain outside the swept path of tall vehicles (buses and trucks circulate on this corridor), with one camera per traffic direction mounted along the arm and angled downward toward its respective lane, both cabled back to a single roadside enclosure housing the processing hardware. This fusion step, and the mounting infrastructure it would require, have not been designed in detail beyond this concept and are identified as future work rather than a capability of the present system.

The seventh limitation is the regulatory positioning of the system as a presumptive violation detection prototype, not as a certified cinemometer. Certified enforcement under Peruvian metrological standards (NMP 013:2010) requires radar or laser sensors with periodic INACAL certification, which is beyond the scope of the current implementation.

The eighth limitation is the pending integration of the traffic signal violation module (Code M30) into the real-time streaming pipeline. The module code was successfully installed and tested on Jetson .27, confirming the correctness of the modular arithmetic approach for fixed-cycle traffic light tracking. However, its integration with the operational streaming pipeline is under development at the time of writing this article.

The ninth limitation is the manual system startup procedure. The current deployment requires manual initiation of the Python scripts after each Jetson boot; implementation of systemd services for automatic startup is recommended to enable unattended operation.

The tenth limitation concerns the non-uniform metric resolution of the homography across the measurement zone, a direct consequence of the oblique camera geometry. Near the camera (L2, y≈665), each pixel corresponds to approximately 2.6 cm of ground distance; in the far zone (near L1, y≈94), this degrades to approximately 33 cm per pixel, a more than twelve-fold reduction in metric resolution. At the aerial camera’s 25 FPS and a vehicle speed of 30 km/h, the frame-to-frame displacement in the far zone is approximately 33 cm, i.e., close to a single pixel. Under these conditions, footpoint detection noise on the order of one pixel is no longer negligible relative to the true displacement, and because speed is computed from displacement magnitude (Equation (3)), such noise biases the estimated speed upward rather than averaging out, increasing the risk of false-positive violation flags for vehicles observed predominantly in the far zone. This effect motivated the definition of the 0–42 m reliable operating zone (Section 3.2.3), which excludes the region beyond the calibrated span where this degradation is most severe; measurements originating outside this zone should be interpreted with reduced confidence.

The eleventh limitation concerns the detection-dataset train/validation/test partition (Section 3.3.1), which stratifies by class label at the individual-image level but does not group images by capture session or vehicle identity. The images were captured as discrete snapshots across separated time windows rather than extracted from continuous video at a fixed interval, which reduces but does not eliminate the possibility that visually near-duplicate observations of the same vehicle were assigned to different partitions. No video- or vehicle-level grouping constraint was applied, so this evaluation protocol may provide a modestly optimistic estimate of generalization to genuinely novel vehicles and scenes; a group-aware re-split (e.g., by capture session) is identified as a methodological refinement for future work.

### 5.4. Implications for Developing Cities

The demonstrated feasibility of the proposed system carries broader implications for road safety in Andean intermediate cities and similar developing-country urban contexts. Table 19 summarizes the incremental hardware cost of the two edge computing nodes. The incremental hardware investment for the edge computing nodes was US$2354 for two Jetson Orin NX 16GB modules. The surveillance cameras, network infrastructure, and pole-mounted support arms were pre-existing from a prior traffic monitoring deployment [25]. Monthly connectivity is approximately US$17 for a 100 Mbps fiber-optic plan including the ONT/router equipment. Importing specialized computing hardware to Peru incurs approximately 29% above FOB prices due to customs duties, nationalization fees, and the 18% IGV tax—a structural cost factor for replication in developing countries. The total system cost remains substantially below that of certified radar-based cinemometers.

The transferability of the calibration procedure, based on drone orthophotography and repeatable topographic survey, allows deployment at any new intersection through a well-defined workflow, without requiring specialized calibration teams. The integration of the Peruvian regulatory framework (DS 025-2021-MTC with the DS 011-2026-MTC modification) as an in-system module aligns the technical output of the system with the administrative requirements for the eventual formalization of the detected violations.

The transparent reporting of methodological findings, including the negative results in the plate detection comparison and the transition from segmentation to detection driven by edge constraints, provides actionable guidance for researchers and practitioners facing similar deployment challenges in developing regions.

## 6. Conclusions

This work presented a distributed edge computing system for real-time urban traffic monitoring, integrating vehicle speed estimation and automatic license plate recognition on two NVIDIA Jetson Orin NX modules. The system was designed, developed, and deployed at Jr. Carlos F. Vivanco, Ayacucho, Peru (2719–2725 m a.s.l.), addressing the enforcement gap that characterizes intermediate Andean cities where certified radar infrastructure is unavailable.

Taken together, the results validate the system’s core premise: accurate, continuous speed and plate measurement is achievable on low-cost edge hardware without certified radar infrastructure. The 40-pass nocturnal campaign against a 1 Hz GNSS Doppler reference is the strongest evidence for this claim. Under the constant-velocity filter deployed during the campaign, the mean absolute error at the enforcement line was 3.19 km/h, with the residual traced to the steady-state lag of that motion model under vehicle acceleration (r=−0.727 with acceleration). Replacing it with a constant-acceleration filter reduced the error to 1.32 km/h under leave-one-pass-out cross-validation and removed the acceleration dependence entirely (r=+0.003), an improvement independently verified on the deployment hardware. The same campaign cross-checked the calibration itself, with longitudinal position agreeing with the GNSS track to a bias of +1.14 m over a 41 m zone.

This accuracy was reached only after diagnosing and correcting three field failure modes that would otherwise have gone unreported: an undetected camera RTSP resolution renegotiation, corrected with a resolution-enforcement guard; a speed-display defect that left stale readings on screen without affecting the logged measurements or violation decisions; and a physical camera displacement between sessions, whose cost was quantified directly (2.42× higher MAE and 3 of 39 violations missed under the stale calibration). Reporting these failure modes alongside the architecture itself, rather than only the post-fix performance, is intended as a practical contribution for groups attempting similar deployments: the distributed two-node architecture (20.0–22.3 and 29–36 FPS), the dual speed-estimation method, the YOLO26n+CCT plate pipeline, and the DS 025-2021-MTC/DS 011-2026-MTC violation classifier are each described in Section 3 together with the conditions under which they were found to fail.

An important methodological finding is the demonstrated equivalence between the model trained exclusively on local Peruvian data (M6) and the model trained on the combined dataset with external Roboflow sources (YOLO26n_suelo). This negative result, transparently reported, indicates that the domain gap between the international plate formats and the standardized Peruvian format outweighs the benefit of additional training volume for this specific application.

Future work is structured along the following lines: first, re-validation of the corrected pipeline (following the resolution-enforcement fix reported in Section 4.3) across an extended campaign of at least 50 passes covering speeds of 0 to 60 km/h, building on the 24-pass campaign reported in this work; second, a Levenshtein-distance-based post-processor for consolidating duplicate plate readings and correcting OCR confusions; third, the replacement of the smartphone frontal camera with a wired IP camera to eliminate WiFi dependency; fourth, complementary training with nighttime data to improve vehicle classification under low-illumination conditions; fifth, the integration of the traffic signal correlation module into the real-time streaming pipeline; sixth, the automatic generation of presumptive violation tickets in PDF format; seventh, the implementation of automatic startup through systemd services for unattended operation; eighth, the validation of the system at a second intersection (Puente Nuevo) to evaluate the transferability of the approach; and finally, cross-correlation between the speed and plate subsystems to link speed measurements with vehicle identification.

The demonstrated feasibility of the proposed system, combined with the estimated cost accessibility relative to certified radar infrastructure and the transferability of the calibration procedure, positions the approach as a scalable and cost-effective solution for smart city applications in developing regions. The transparent reporting of results, limitations, and methodological findings aims to contribute to the emerging body of literature on the practical deployment of intelligent transportation systems in real operational contexts.

## Figures and Tables

**Figure 1:**
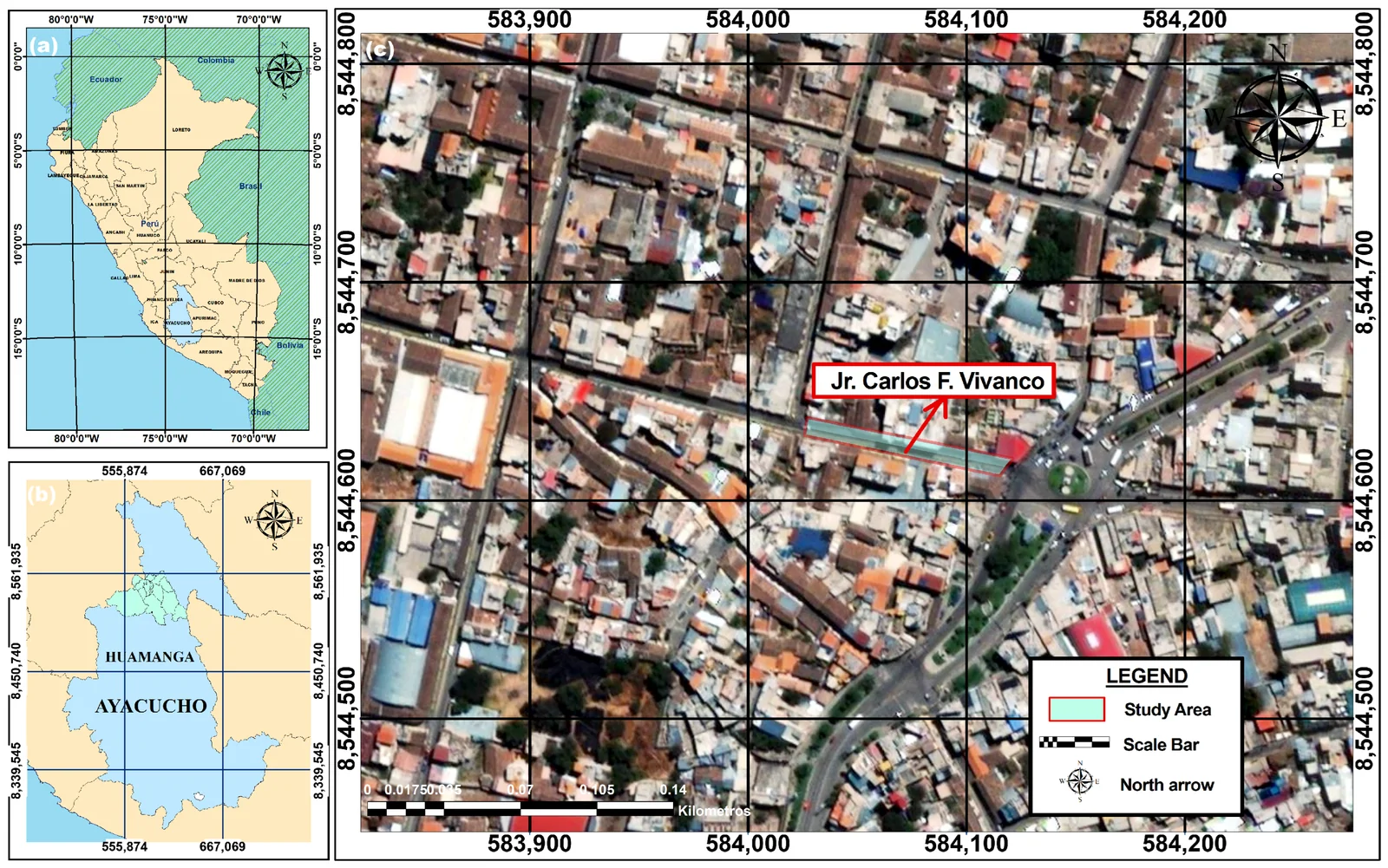
Study area. (**a**) Location of Peru with the Ayacucho department highlighted. (**b**) Huamanga province showing the Ayacucho district. (**c**) Satellite view of Jr. Carlos F. Vivanco showing the monitored traffic segment. Coordinate values correspond to UTM projected coordinates (WGS84, Zone 18S). Panels (**a**,**b**) were adapted from [27].

**Figure 2:**
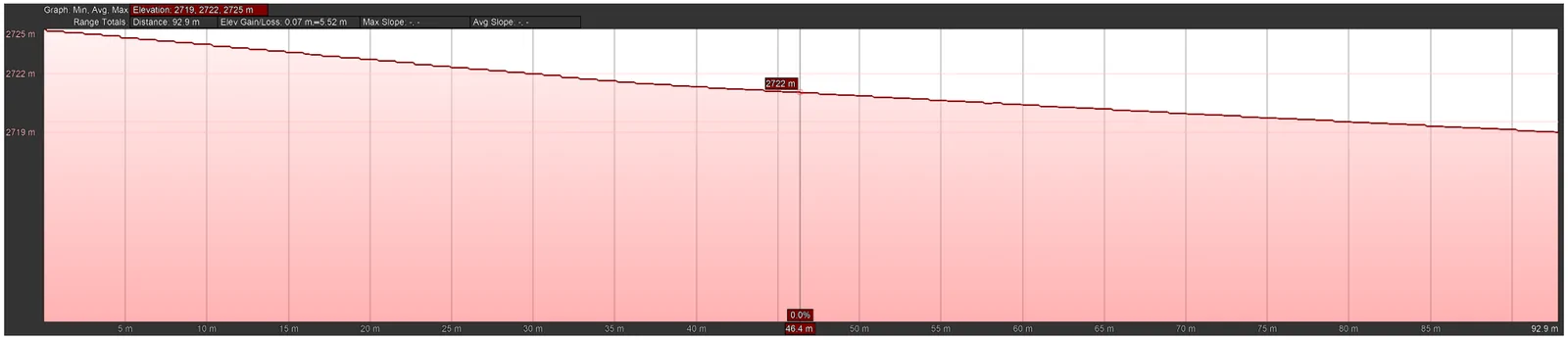
Elevation profile of the study segment obtained from Google Earth Pro. The profile confirms a predominantly linear descending slope of 5.94% (elevation loss of 5.52 m over 92.9 m, from 2725 to 2719 m a.s.l.). The combined slope and UTM grid-to-ground correction factor $f_{s}=1.0025$ is applied to both the homography-based and line-crossing speed estimation methods.

**Figure 4:**
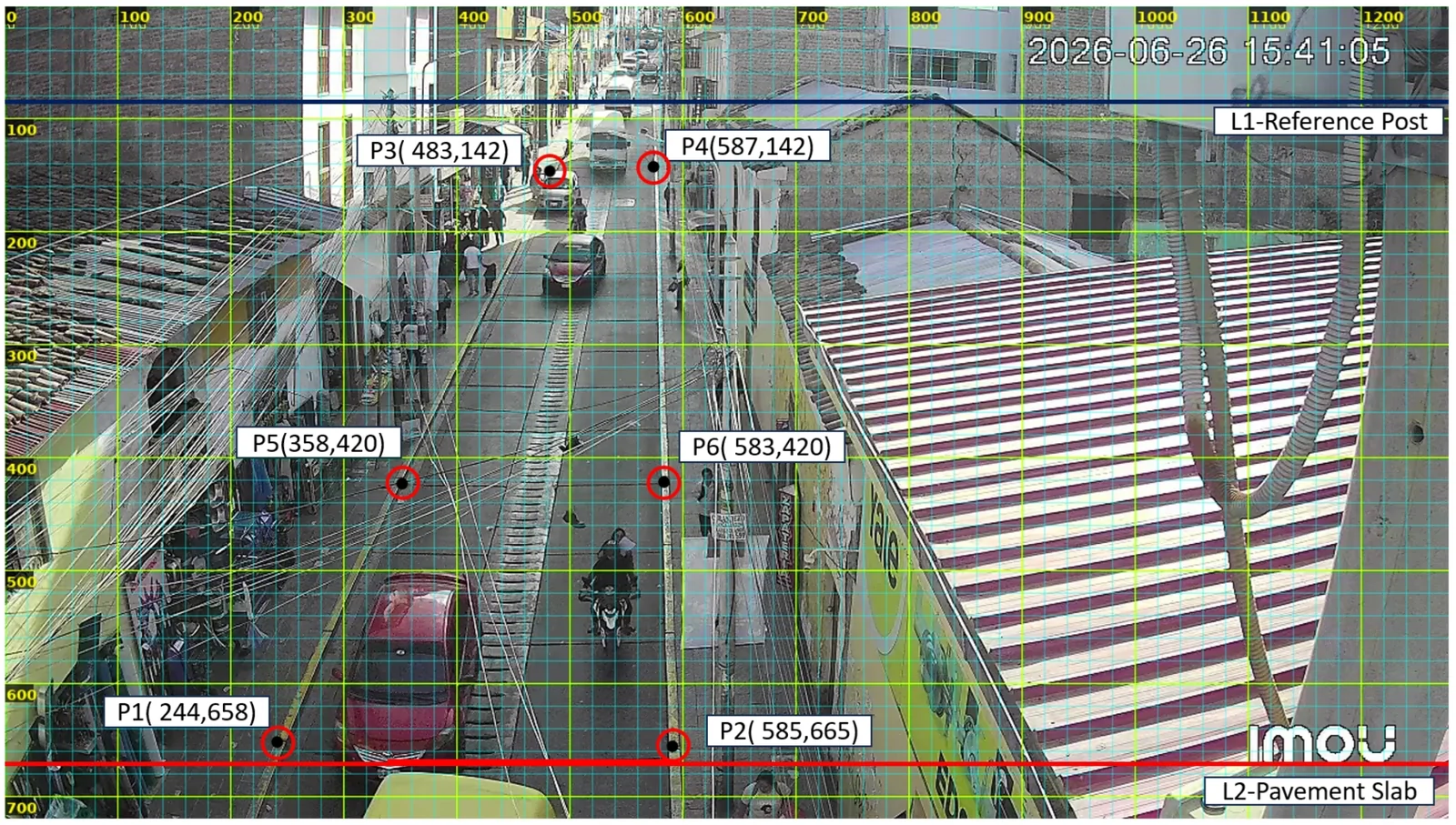
Camera calibration frame from the IMOU aerial camera (1280 × 720 px, 25 fps) with pixel coordinate grid overlay. The six homography calibration points (P1–P6) are identified at their pixel coordinates (u,v), corresponding to the metric positions shown in Figure 3. The horizontal blue line indicates L1 (Reference Post, y=94 px) and the horizontal red line indicates L2 (Pavement Slab, y=665 px).

**Figure 5:**
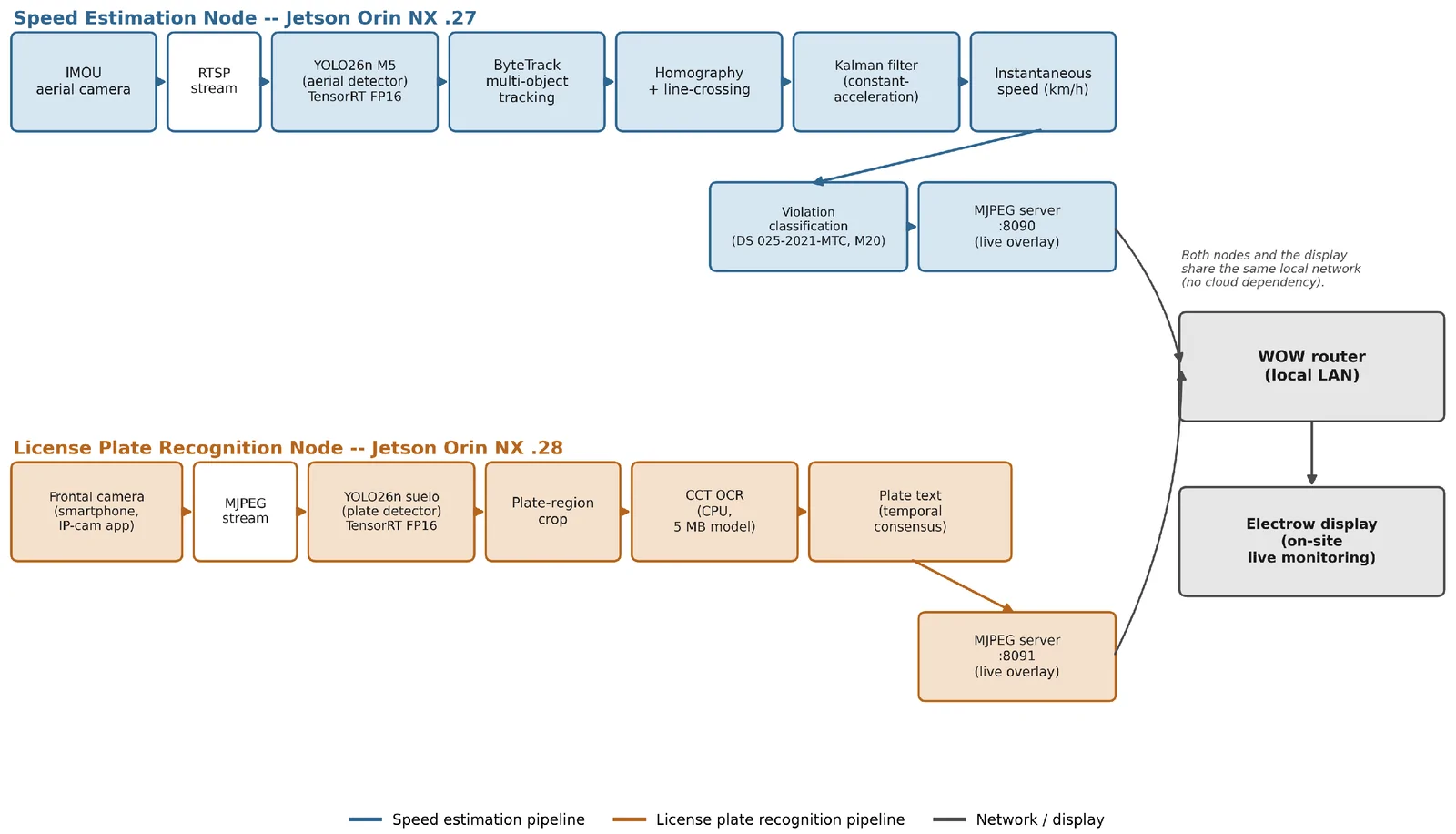
System architecture showing the two-node edge computing pipeline, network topology, and data flows.

**Figure 6:**
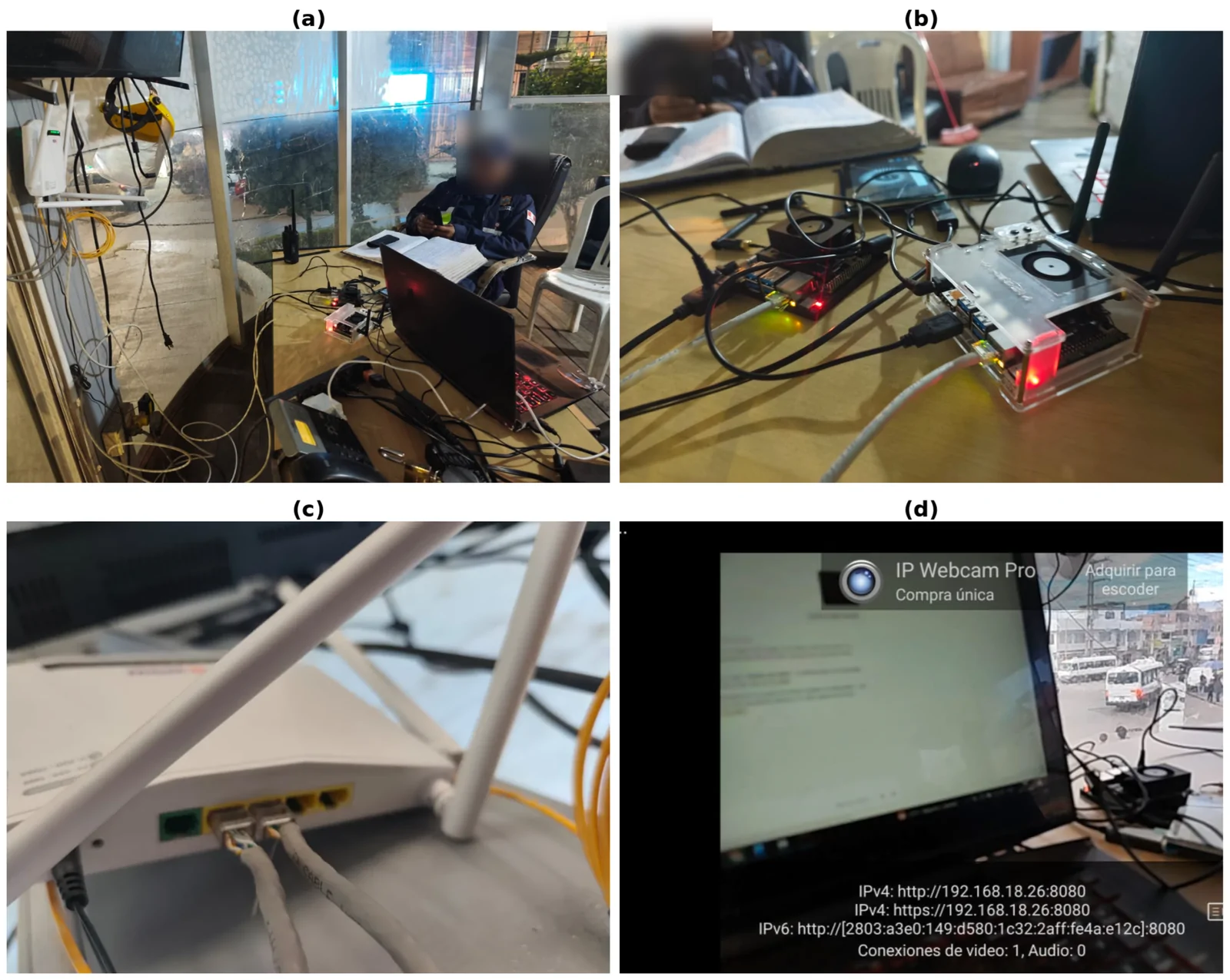
Physical deployment of the traffic monitoring system in the Serenazgo (municipal security) booth on Jr. Carlos F. Vivanco. (**a**) Overall view of the booth showing the WOW ATW-624G router mounted on the wall (top left), a Jetson Orin NX on the desk, the monitoring laptop, and Ethernet cabling. (**b**) Close-up view of the two NVIDIA Jetson Orin NX modules (16 GB each) with active cooling fans, WiFi antennas, and Ethernet connections. (**c**) Rear view of the WOW ATW-624G router showing the Ethernet cables connected to the LAN ports. (**d**) Smartphone running the IP Webcam Pro application, serving as the frontal camera for the license plate recognition subsystem, with one active video connection.

**Figure 7:**
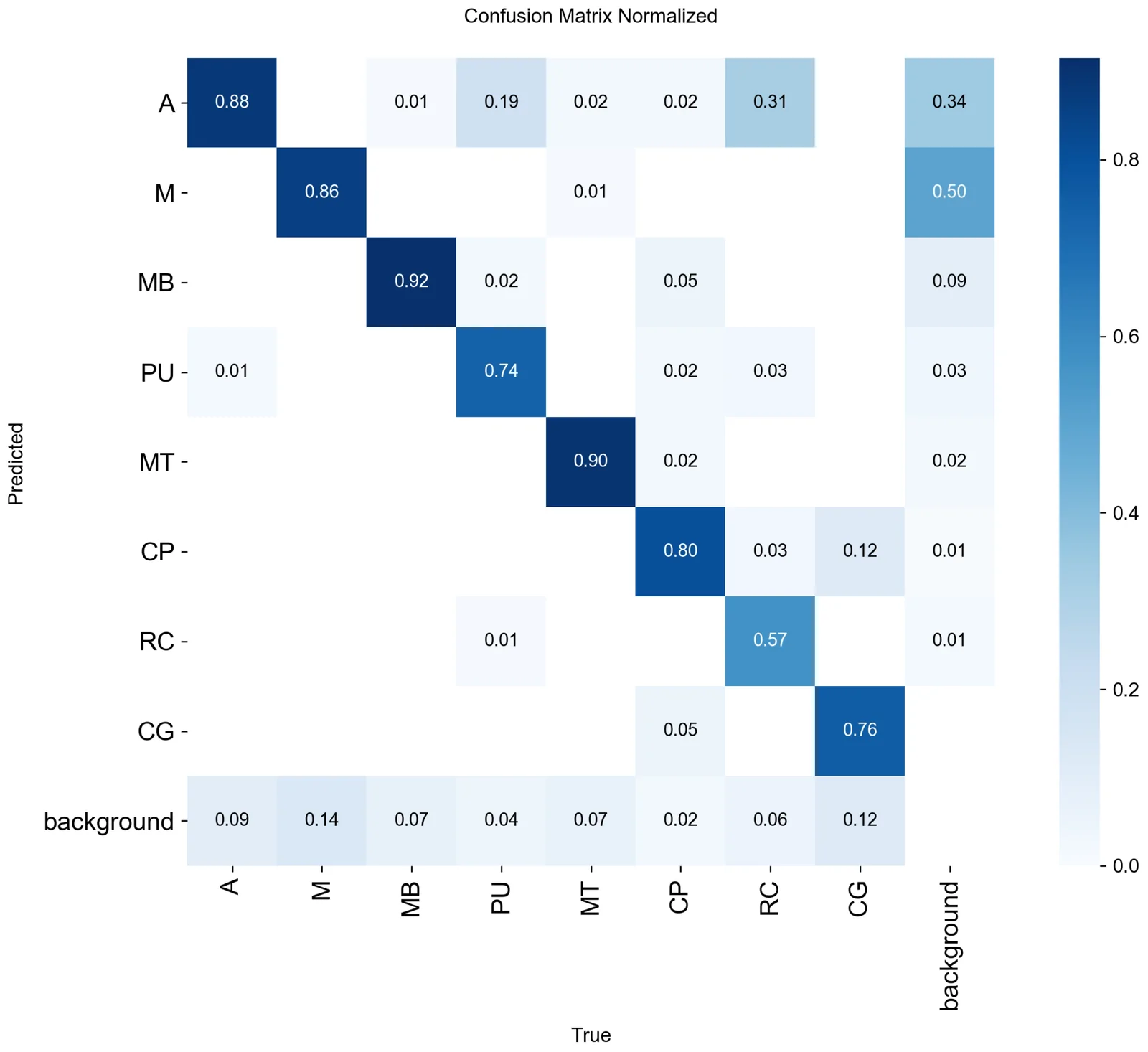
Normalized confusion matrix for the M5 detection model (YOLO26n-det) on the held-out test set. Rows are predicted classes, columns are ground-truth classes; values are row-normalized fractions. The 0.31 entry for RC (true) predicted as A quantifies the automobile/rural-combi confusion discussed in the text; the “background” column/row reflects missed detections and spurious background predictions, respectively.

**Figure 8:**
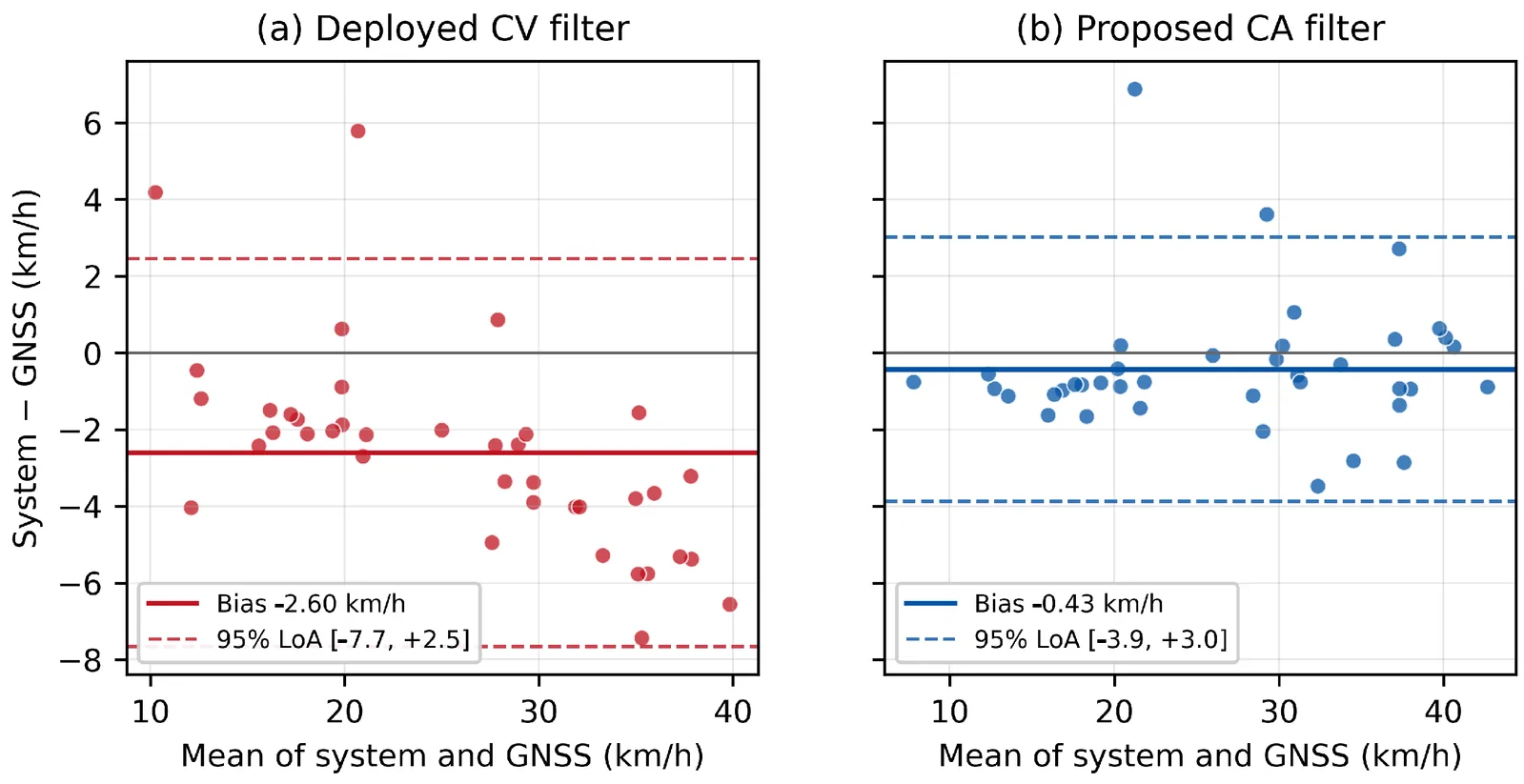
Bland–Altman agreement between the system and the GNSS Doppler reference at the enforcement line, one reading per pass (N=39). (**a**) Constant-velocity filter as deployed: bias −2.60 km/h, limits of agreement [−7.7,+2.5] km/h, with the residual becoming more negative at higher speed. (**b**) Proposed constant-acceleration filter: bias −0.43 km/h, limits of agreement [−3.9,+3.0] km/h, with no trend against the mean.

**Figure 9:**
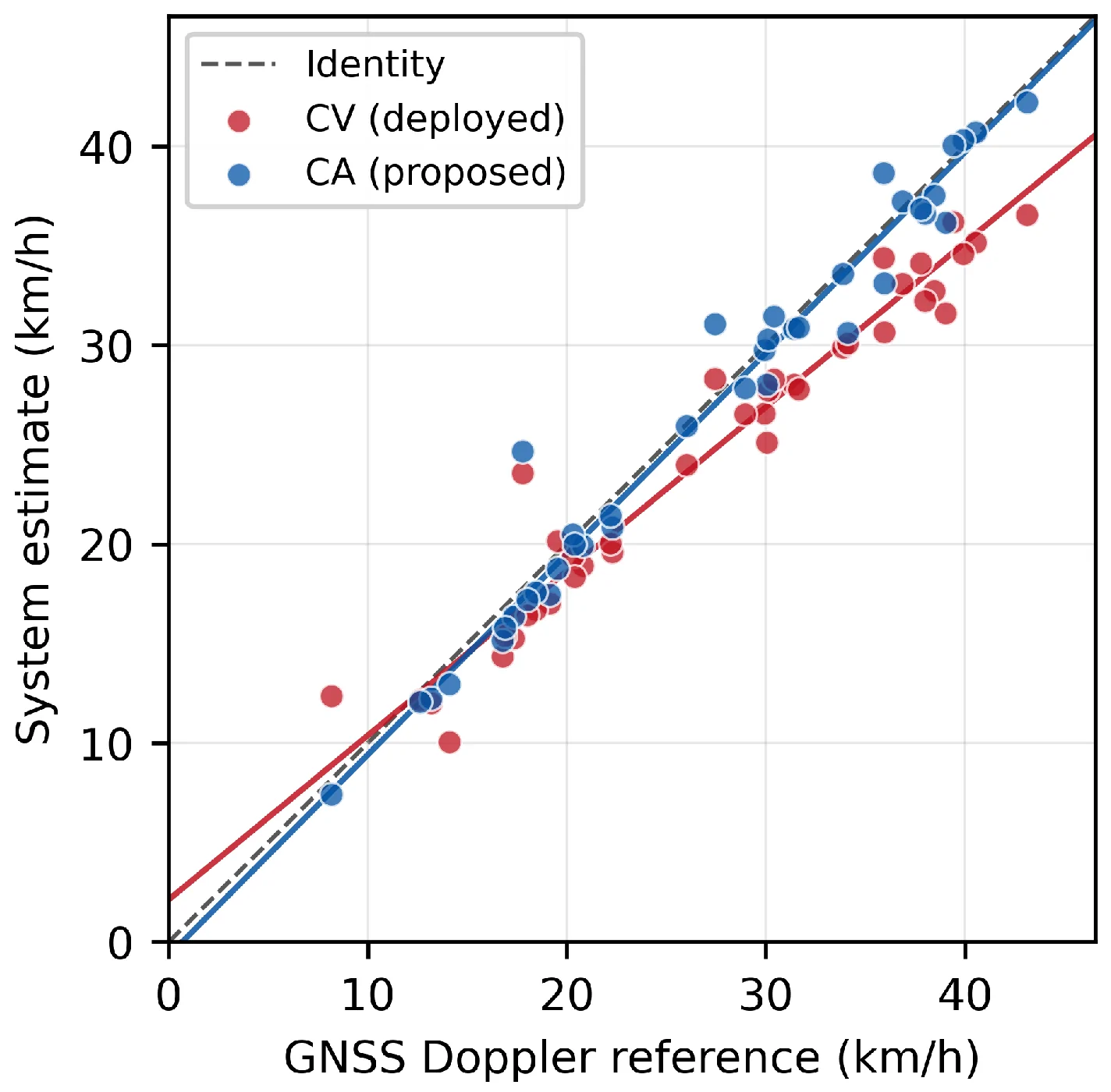
System estimate against the GNSS Doppler reference at the enforcement line, with the identity line (dashed) and the Deming fit for each estimator. The deployed constant-velocity filter has a Deming slope of 0.825; the proposed constant-acceleration filter, 1.009. Deming rather than ordinary least-squares regression is used because both variables carry measurement error.

**Figure 10:**
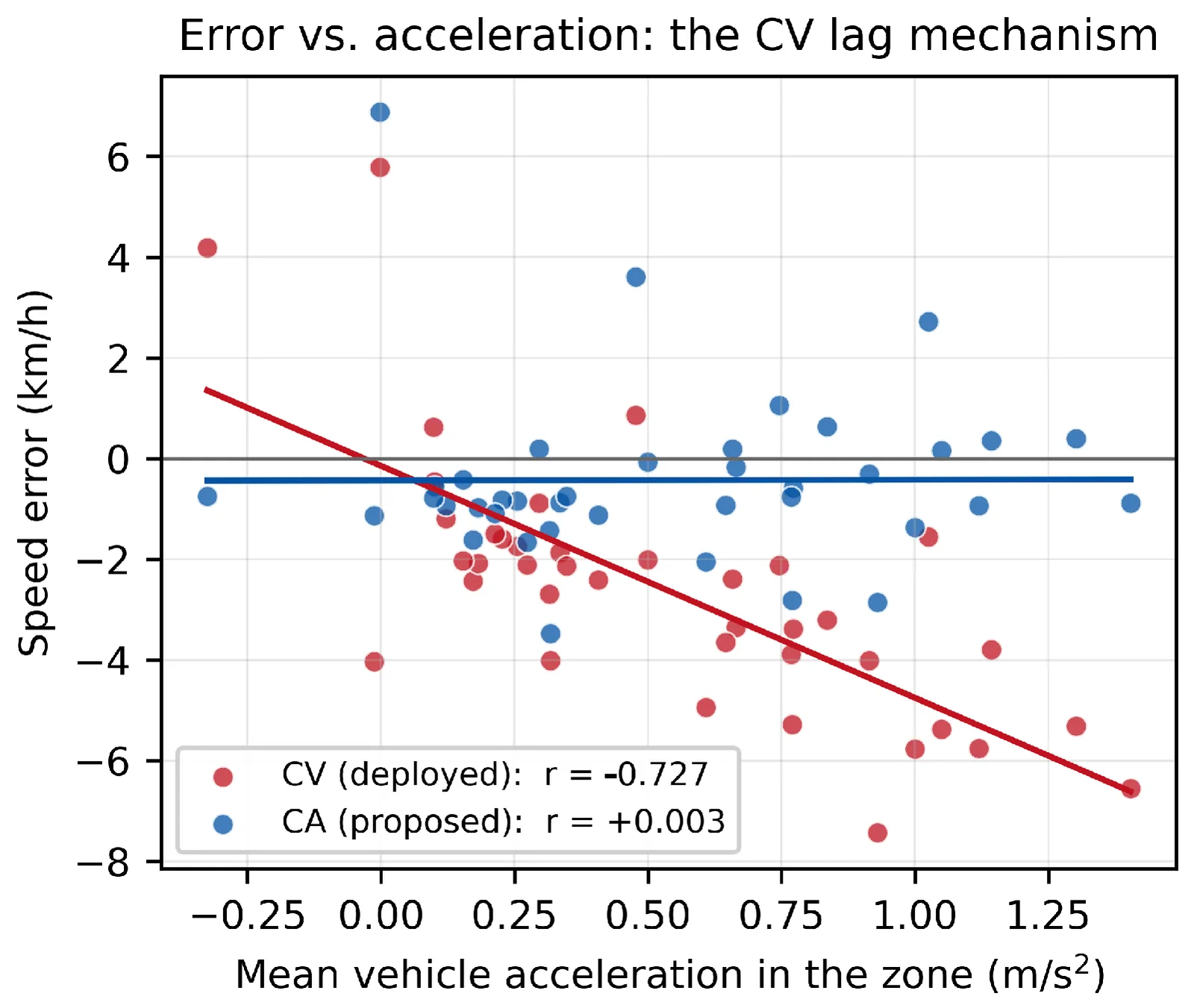
Speed error against the mean acceleration of the vehicle within the measurement zone, one point per pass. The deployed constant-velocity filter shows the steady-state lag expected of a constant-velocity model tracking an accelerating target (r=−0.727; −4.61 km/h per m s^−2^). Adding the acceleration term to the state removes the dependence entirely (r=+0.003).

**Figure 11:**
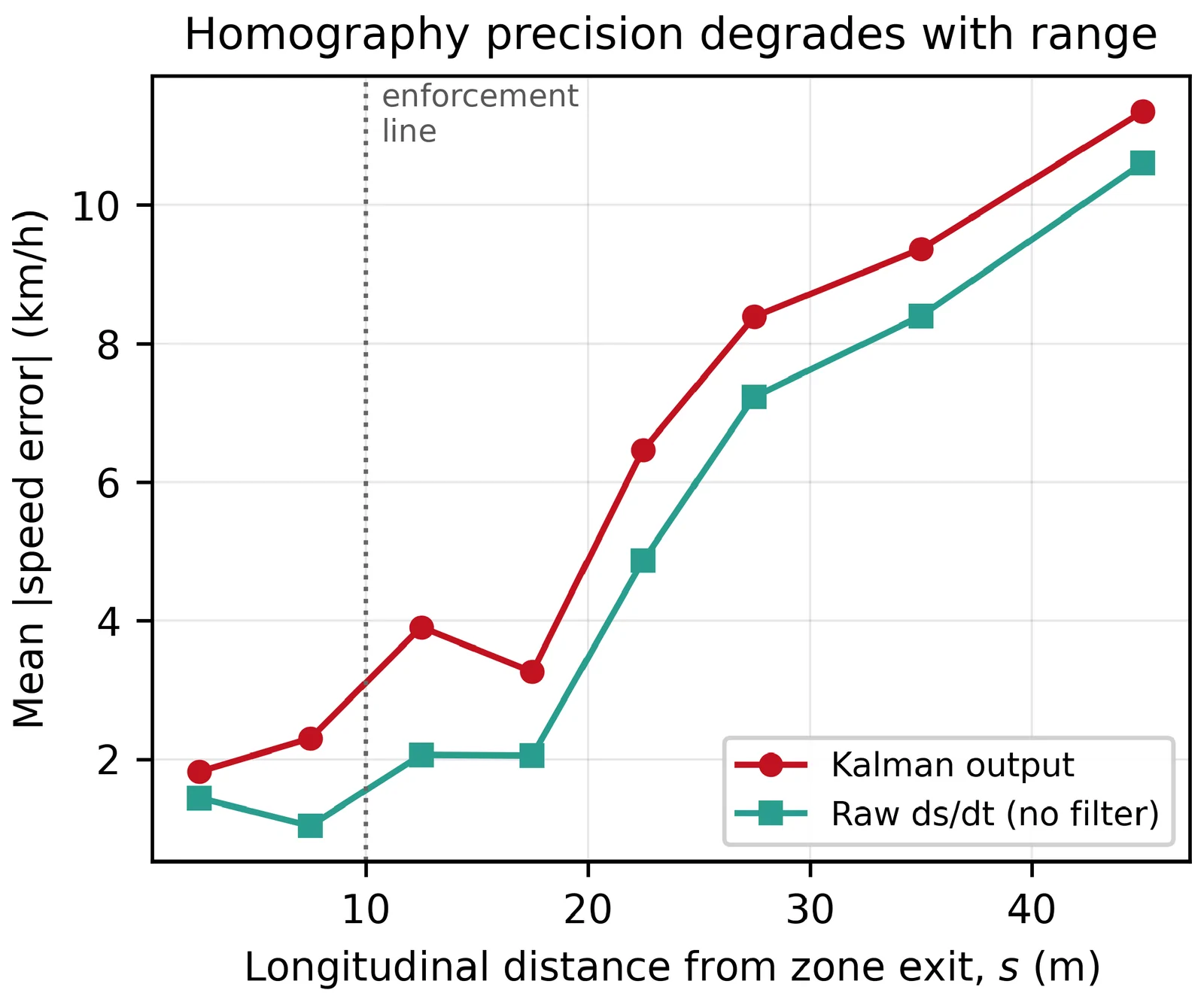
Mean absolute speed error as a function of longitudinal distance from the zone exit. The unfiltered position derivative follows the same trend as the filtered output, which identifies range dependence as a property of the projective geometry rather than of the filter. The dotted line marks the enforcement line at s=10 m.

**Figure 12:**
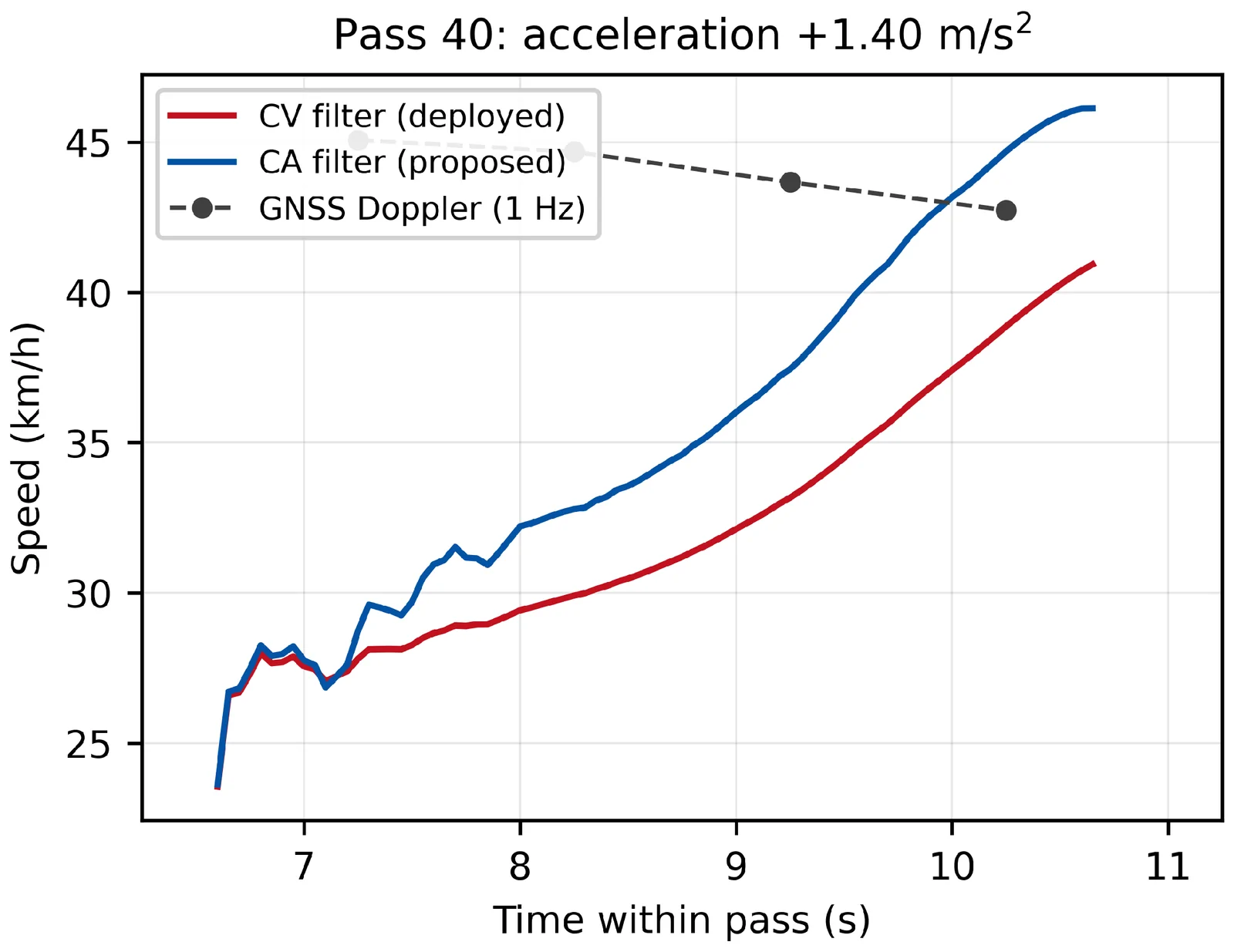
Speed profile of the pass with the highest mean acceleration, comparing the GNSS Doppler reference with the deployed constant-velocity filter and the proposed constant-acceleration filter. The constant-velocity estimate trails the reference throughout the traverse, which is the behaviour quantified in Figure 10.

**Figure 13:**
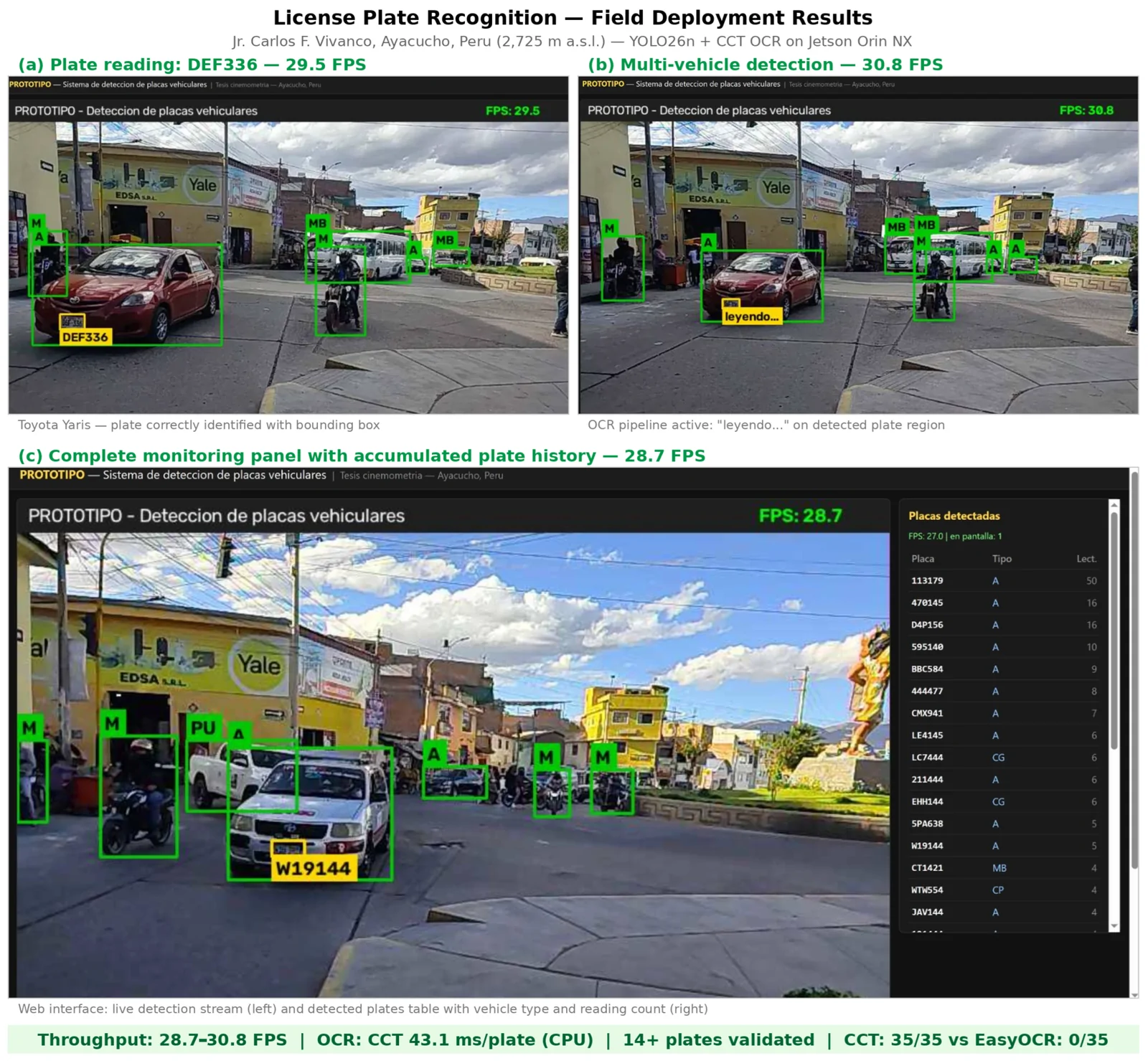
License plate recognition on the operational system. (**a**) Detection and recognition of plate DEF336 on a motorcycle, showing the vehicle bounding box, plate region, and OCR reading. (**b**) System status panel showing the plate reading in progress with real-time character extraction. (**c**) Complete monitoring panel displaying the aggregated recognized plate W19144 with vehicle classification and tracking ID. The panels illustrate the multi-threaded pipeline (Section 3.5.4) operating at 29–36 FPS on the Jetson Orin NX node.

**Figure 14:**
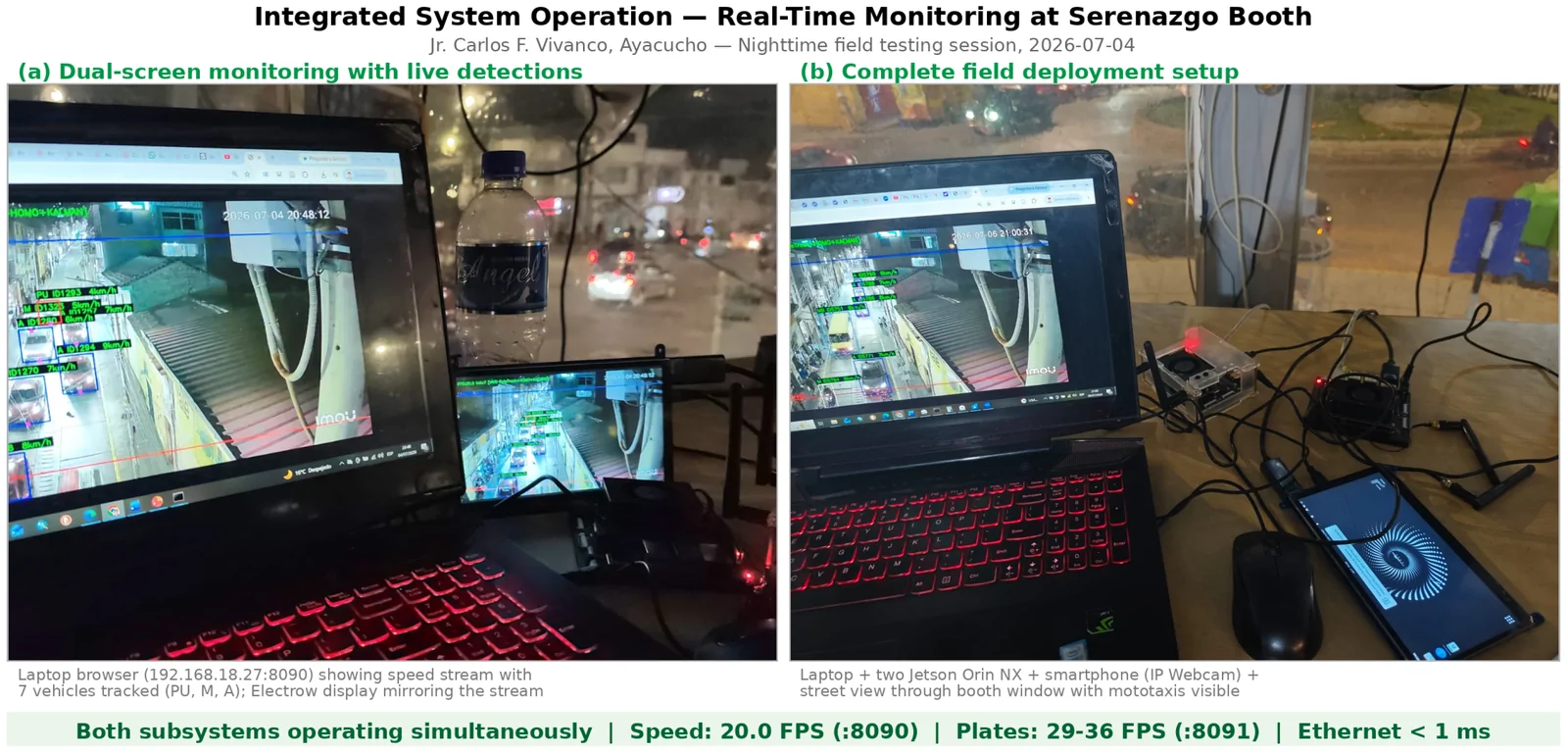
Integrated operation of the deployed system. (**a**) Dual-screen monitoring showing the speed estimation dashboard (**left**, Jetson .27) and the license plate recognition dashboard (**right**, Jetson .28) operating simultaneously on the Serenazgo booth display. (**b**) Complete system installation showing the physical arrangement of both Jetson modules, the WOW ATW-624G router, the Electrow monitoring display, and the smartphone frontal camera.

**Figure 15:**
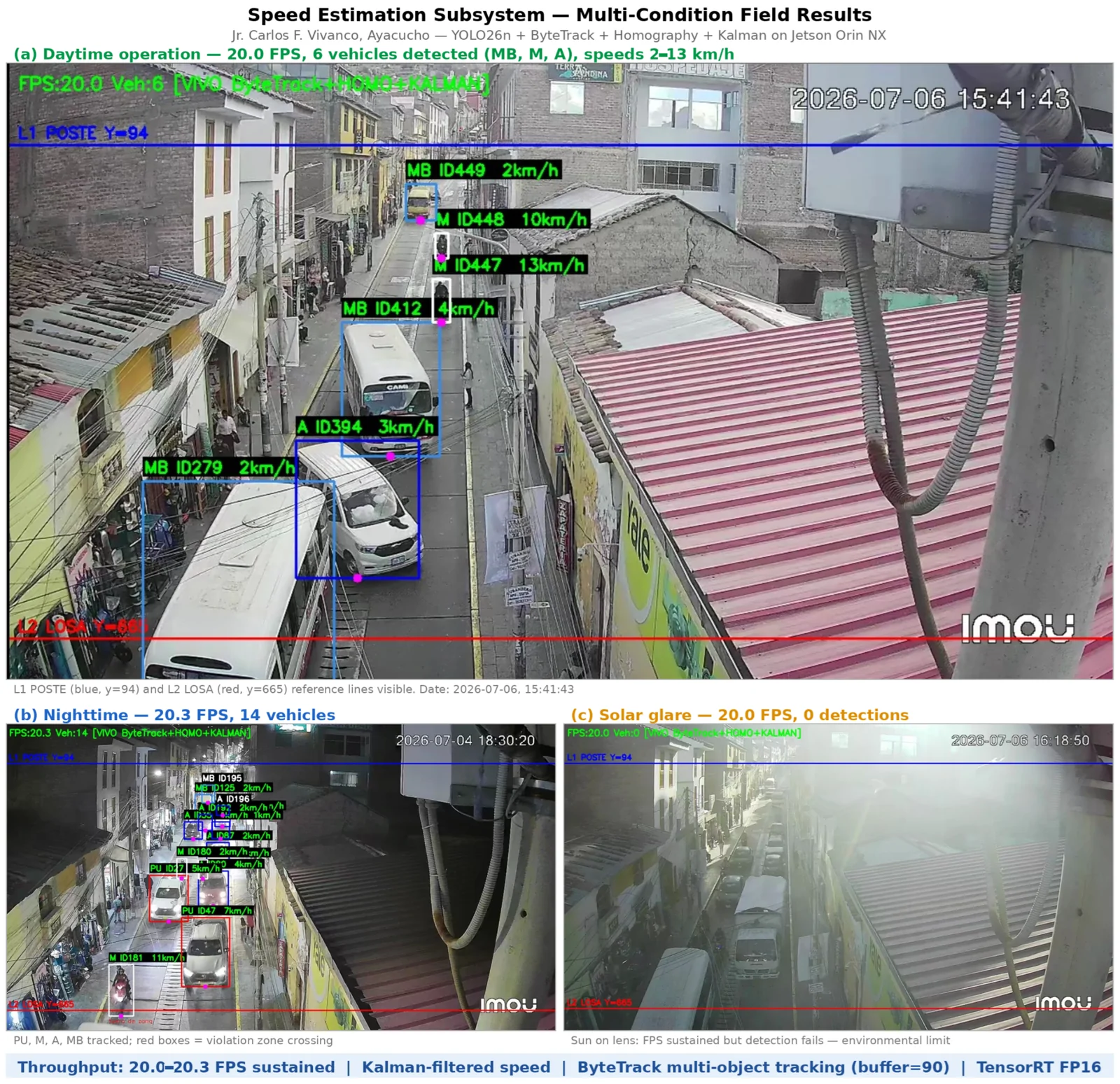
Speed estimation under three representative operational conditions. (**a**) Daytime operation with clear visibility, showing multiple vehicles tracked simultaneously with individual speed estimates. (**b**) Nighttime operation under yellow sodium street lighting, illustrating the sustained detection capability despite reduced contour visibility. (**c**) Daytime operation with sun halo interference on the camera lens, illustrating a documented failure mode in which direct sun halo on the camera lens causes temporary detection loss; the ByteTrack persistence filter recovers tracking when the artifact clears within approximately 2–3 s. In all panels, the visible line overlays correspond to L1 (blue, upper) and L2 (red, lower) used by the complementary line-crossing method.

**Table 1:** Comparative positioning of the proposed system against related work. ✓ = feature present; — = feature absent. Accuracy/error and test sample size (*N*) are reported exactly as stated in each source; “n.r.” indicates the source does not state an explicit *N* for the corresponding accuracy figure.

System	Speed	ALPR	Edge	Violation	Field Valid.	Accuracy/Error	*N* (Test)
Lin et al. [8]	✓	—	—	—	✓	7.6% MAPE	17 vehicles
Merrad et al. [7]	✓	—	—	—	✓	<3% (avg. 2.29%)	2 vehicles
Lian et al. [12]	✓	—	—	—	✓	95.1–97.6% accuracy	n.r. ^*a*^
Kesli and Kilicarslan [35]	✓	—	✓	—	✓	MAE 1.34 km/h	n.r. ^*b*^
Ammar et al. [20]	—	✓	✓	—	✓	74% plates, 43% model	119 vehicles ^*c*^
Meesad and Thumthong [21]	—	✓	✓	—	✓	99.16% accuracy	10,000 img. + 2000 clips
Ashraf et al. [38]	✓	—	—	—	✓	87.242% (ISEP)	n.r.
Islam et al. [31]	✓	—	✓	—	✓	MAE 3.2 km/h	n.r.
Proposed system	✓	✓	✓	✓	✓	3.19/1.32 km/h ^*d*^	40 ^*d*^

^*a*^ The source states only that “tens of thousands of experiments” were conducted overall, without an *N* tied specifically to the 95.1–97.6% figure. ^*b*^ Per-video test counts are tabulated (six UTFPR videos, two BRNO videos) but no single summed *N* is stated for the combined MAE. ^*c*^ 119 unique vehicles observed across two videos (32 min 46 s total); a separate static-image evaluation used 100 images and 1382 characters. ^*d*^ Mean absolute error at the enforcement line over the 40-pass nocturnal campaign of 18–19 September 2026 (1 Hz GNSS Doppler reference, 16.9–48.8 km/h; 39 of the 40 passes yielded a valid reading at the line). The first figure is the configuration deployed during the campaign (constant-velocity Kalman filter); the second is the proposed constant-acceleration filter under leave-one-pass-out cross-validation, verified to transfer to the deployment hardware (Section Equivalence Between Offline Reprocessing and the Deployed Edge Hardware). Two earlier and methodologically non-comparable estimates (N=3, 0–14 km/h, 6 July 2026; N=2, 16–22 km/h, 15 August 2026) are reported in Section 4.3 for completeness.

**Table 2:** Distance measurements obtained through three independent methods for the study segment on Jr. Carlos F. Vivanco.

Method	Distance (m)	Measurement Type	Precision
Tape measure (field)	∼92–93	Inclined surface	±10–20 cm
Google Earth Pro	92.9	Horizontal projection	±1–3 m
Drone + AutoCAD Civil 3D 2025	84.1 (L1–L2)	Calibrated horizontal	±10 cm

**Table 4:** Class distribution in the aerial dataset after cleaning (2985 images, 44,505 instances). The distribution reflects the observed vehicular frequency in the Ayacucho historic center, where automobiles and motorcycles predominate due to the narrow colonial streets.

Class	Description	Instances	%
A	Automobiles/Station wagons	18,565	41.7
M	Motorcycles/Bicycles	15,785	35.5
MB	Microbuses	5522	12.4
PU	Pickup trucks/SUVs	2325	5.2
MT	Mototaxis	1286	2.9
CP	Small trucks (C2/C3)	422	0.9
RC	Rural combis/Panels	359	0.8
CG	Large trucks (C4/8 × 4)	241	0.5
Total		44,505	100.0

**Table 5:** External data sources combined into DATASET_SUELO. “Raw size” is the total number of images available in the source dataset; “Used” is the number actually contributed after filtering/subsampling.

Source	Provider (License)	Raw Size	Used	Selection Criterion
QHAWAY (remapped)	De la Cruz-Moran et al. [27], local Ayacucho collection	1340	1135	Class-relevance filter: images kept only if they contain ≥1 instance of automobile/bus/truck/mototaxi; 205 images with only non-target classes discarded
License Plate Recognition v11	roboflow-universe-projects [71] (CC BY 4.0), https://universe.roboflow.com/roboflow-universe-projects/license-plate-recognition-rxg4e (accessed on 22 June 2026)	10,125	1998	Random subsample (seed 42), stratified proportionally per split, capped at 2000 images to avoid overrepresentation of this multi-country dataset
Peru License Plate v9	billy-soplin [72] (CC BY 4.0), https://universe.roboflow.com/billy-soplin/peru-license-plate (accessed on 22 June 2026)	2626	2622	Full set used; 4 images dropped for empty/invalid annotations after format conversion
Peruvian plate detection v2	tp2-bbjq4 [73] (MIT), https://universe.roboflow.com/tp2-bbjq4/peruvian-plate-detection-rbdtj (accessed on 22 June 2026)	297	287	Full set used; 10 images dropped for empty/invalid annotations after format conversion
Total (4 sources)		14,388	6042	

**Table 6:** Comparative performance of the six trained models on their respective test sets. Box and Mask mAP values are reported with IoU thresholds of 0.5 and 0.5:0.95. Models M1–M4 were evaluated on the aerial test set (305 images); M5 on the same aerial test set; M6 on the frontal test set (150 images). Dashes indicate metrics not applicable to detection-only models.

Model	Architecture	Task	Imgsz	mAP50 (Box)	mAP50-95 (Box)	mAP50 (Mask)	mAP50-95 (Mask)	P	R
M1	YOLOv8m-seg	seg	640	0.859	0.719	0.824	0.624	0.893	0.798
M2	YOLO11m-seg	seg	640	0.863	0.720	0.829	0.622	0.873	0.816
M3	YOLO26m-seg	seg	640	0.903	0.788	0.857	0.668	0.900	0.850
M4	YOLO26m-seg	seg	1024	0.920	0.811	0.891	0.731	0.927	0.873
M5	YOLO26n-det	det	640	0.856	0.721	—	—	0.878	0.795
M6	YOLO26n-det	det	640	0.845	0.661	—	—	0.895	0.764

**Table 7:** Per-class detection performance of M5 (YOLO26n-det) on the aerial test set (305 images, 4511 instances). F1 is computed as the harmonic mean of Precision and Recall.

Class	mAP50	mAP50-95	P	R	F1
MT (Mototaxi)	0.949	0.858	0.951	0.917	0.933
MB (Microbus)	0.930	0.799	0.944	0.889	0.916
A (Automobile)	0.918	0.756	0.933	0.833	0.880
M (Motorcycle)	0.873	0.617	0.911	0.802	0.853
CG (Large truck)	0.841	0.707	0.868	0.787	0.825
PU (Pickup/SUV)	0.835	0.737	0.885	0.724	0.796
CP (Small truck)	0.803	0.690	0.799	0.780	0.790
RC (Rural combi)	0.695	0.605	0.735	0.629	0.678
All classes	0.856	0.721	0.878	0.795	0.835

**Table 8:** Hardware components of the deployed traffic monitoring system. All wired connections use Cat5e Ethernet cables through the WOW ATW-624G router.

Component	Specification	Function	Connection
Jetson Orin NX (.27)	16 GB LPDDR5, 157 TOPS	Speed estimation	Ethernet (<0.75 ms)
Jetson Orin NX (.28)	16 GB LPDDR5, 157 TOPS	License plate recognition	Ethernet (<0.88 ms)
IMOU camera (.22)	1280 × 720, 25 fps, RTSP	Aerial view	Ethernet
Smartphone + IP Webcam	800 × 600, 30 fps, MJPEG	Frontal view	WiFi 5 GHz
WOW ATW-624G router	5/2.4 GHz, 4 × GbE LAN	Local network	—
Electrow display	1024 × 600, 10.1”	Monitoring dashboard	DisplayPort

**Table 9:** Sensitivity of the reported metrics to the video–GNSS temporal alignment. The correction is derived from position and does not improve the speed agreement.

Alignment	Position	Position	Speed	Speed
	Bias (m)	RMSE (m)	RMSE (km/h)	Bias (km/h)
Uncorrected	−6.20	7.50	4.71	−3.38
−1.75 s	−12.99	14.88	4.47	−2.86
+1.75 s (adopted)	+0.98	3.83	5.50	−3.54

**Table 10:** Speed violation classification per DS 025-2021-MTC (Code M20). UIT 2026 = S/ 5500. The camera tolerance of 5 km/h (DS 011-2026-MTC) is applied prior to classification.

Code	Excess Range	Fine (% UIT)	Points
M20-a	Δv≤10 km/h	18% (S/ 990)	50
M20-b	10<Δv≤30 km/h	24% (S/ 1320)	60
M20-c	Δv>30 km/h	50% (S/ 2750)	70

**Table 11:** Reprojection error of the six homography calibration points, regenerated directly from the deployed homography matrix (Equation (7)). The Euclidean error is computed as the distance between the pixel-to-metric transformed point and the ground-truth metric coordinate. The longitudinal error is the projection of the Euclidean error vector onto the near-to-far baseline axis defined above.

Point	Euclidean Error (cm)	Longitudinal Error (cm)	Position
P1	18.5	8.6	Near L2, left
P2	29.7	1.6	Near L2, right
P3	18.4	2.7	Far L1, left
P4	14.2	0.3	Far L1, right
P5	11.4	11.4	Mid-depth, left
P6	17.7	1.7	Mid-depth, right
Mean	18.3	4.4	—

**Table 12:** Speed estimators compared at the enforcement line (s=10 m; N=39 passes). All estimators are causal except where noted. MAE_LOO_ is the leave-one-pass-out cross-validated error, in which the tuning parameter is chosen without reference to the pass being evaluated.

Estimator	MAE	MAE_LOO_	Bias	Deming	CCC
	(km/h)	(km/h)	(km/h)	Slope	
CV filter, as deployed ($\sigma_{a}=1.5$ m s^−2^)	3.19	—	−2.60	0.825	0.915
CV filter, inflated process noise	1.31	1.40	−0.43	0.932	0.982
Causal backward-window derivative	1.44	1.36	−0.78	0.928	0.980
CA filter (proposed)	1.26	1.32	−0.43	1.009	0.982

Centred-window derivative (non-causal; upper bound only): MAE 1.33 km/h.

**Table 13:** Equivalence between offline reprocessing and the deployment node, over the same ten passes. The quantity of interest is the difference between rows, not the absolute error level (see text).

Configuration	Detections	MAE, CV	MAE, CA	Bias, CA
		(km/h)	(km/h)	(km/h)
Workstation, FP32, all frames	23,896	3.64	0.64	−0.54
Jetson, FP16, all frames	23,395	3.63	0.64	−0.55
Jetson, FP16, 80% frame coverage	18,683	3.55	0.68	−0.63

Paired difference (Jetson − workstation), CA reading: mean −0.02 km/h, SD 0.08, max |Δ| 0.17 km/h.

**Table 14:** Comparison of plate detection models. Both models were evaluated on the same 150-image proprietary test set. The plate-class metric is the primary performance indicator for the target application.

Model	Train Images	Plate mAP50	Overall mAP50	Size
M6 (proprietary only)	1513	0.815	0.845	5.4 MB
YOLO26n_suelo (combined)	7555	0.807	0.851	5.4 MB

**Table 15:** Peruvian license plates correctly recognized by the operational system during field testing on 6 July 2026 (nighttime). All plates were validated by manual inspection of the corresponding video frames.

Plate	Vehicle Type	Time	Plate	Vehicle Type	Time
C3E040	Automobile	20:15	BHW576	Automobile	20:41
TAL876	Automobile	20:18	BWW590	Microbus	20:44
BLQ468	Microbus	20:22	LFL441	Automobile	20:48
COD598	Automobile	20:26	LE4145	Pickup	20:52
BAM353	Automobile	20:30	AZ5544	Automobile	20:56
BBC584	Automobile	20:34	C8N243	Automobile	21:00
DEF336	Motorcycle	20:38	WB1099	Microbus	21:04

**Table 16:** Sustained real-time performance of both processing nodes measured during a continuous 4-h operation window on 6 July 2026. FPS values are averaged over 1-min intervals and reported as min–max range.

Metric	Jetson .27 (Speed)	Jetson .28 (Plates)
Model	YOLO26n-det (M5)	YOLO26n_suelo + CCT
Model size	7.5 MB (FP16 engine)	7.4 MB + 5.0 MB
Input resolution	1280 × 720	800 × 600
Camera FPS	25	30
Sustained FPS	20.0–22.3	29–36
GPU utilization	78–85%	65–72%
Power draw	12–14 W	10–12 W
Memory (LPDDR5)	4.1 GB	3.8 GB

**Table 17:** Comparison of system performance between daytime and nighttime operation windows. Detection and tracking rates were measured on 200-vehicle samples per condition (ground truth via manual review); confidence intervals for proportions were computed with the Wilson score method.

Metric	Daytime (14:00–17:00)	Nighttime (19:00–21:00)	95% CI (Wilson)
Vehicle detection rate	97.0% (194/200)	92.3% (185/200)	[93.3, 98.5]% day; [87.8, 95.2]% night
Tracking persistence (frames)	87.4 (avg)	74.1 (avg)	n/a
Plate reading success	78.6% (157/200)	71.0% (142/200)	[72.4, 83.7]% day; [64.6, 77.0]% night
False positives (per hour)	2.1	4.3	n/a

**Table 18:** Field log used to assess same-vehicle dual-method coverage: homography-based instantaneous speed versus line-crossing speed for all vehicles tracked in one representative session (n=26).

Metric	Value
Vehicles tracked	26
Vehicles with a homography-based speed reading (>0 km/h)	17/26 (65%)
Vehicles that completed the L1–L2 crossing (cruzo_L2 = yes)	0/26 (0%)
Paired homography/line-crossing measurements for the same vehicle	0

**Table 19:** Incremental hardware cost of the proposed edge computing system.

Component	Specification	Qty	Cost (USD)
Jetson Orin NX 16GB	Edge AI computing node	1	1326 ^*a*^
Jetson Orin NX 16GB	Edge AI computing node	1	1028 ^*b*^
Total new hardware			2354

^*a*^ Imported via DHL; includes FOB (US$1023) plus Peruvian customs duties and nationalization fees (PEN 1121, approximately 29% over FOB). ^*b*^ Purchased via Amazon with shipping and taxes included.

## Data Availability

The calibration scripts, homography parameters, system configuration files, and inference code supporting the reported results are publicly available at https://github.com/diegotenorio16-tech/vehicular-speed-estimation-edge-computing (accessed on 20 August 2026). The trained model weights (.engine files optimized for NVIDIA Jetson Orin NX) are available upon reasonable request to the corresponding author due to platform-specific compilation. The proprietary aerial and frontal datasets contain images captured in a public urban corridor and can be shared under a data usage agreement for research purposes.

## Supplementary Materials

The following supporting information can be downloaded at https://www.mdpi.com/article/10.3390/s26216845/s1, Supplementary Material File S1: full report of the preliminary (6 July 2026) and extended nocturnal (15 August 2026) speed validation campaigns, superseded by the corrected-pipeline campaign of Section 4.3, including validation tables and figures omitted from the main text for length; confirmed track identifiers for the 40-pass corrected-pipeline campaign. Supplementary Material File S2: full per-model training configuration of the four segmentation architectures (M1–M4) evaluated on the aerial dataset, superseded by the detection-only models (M5–M6) deployed on the edge hardware, including individual per-architecture training detail omitted from the main text for length.

## Abbreviations

The following abbreviations are used in this manuscript:

ALPR	Automatic License Plate Recognition
CCT	Compact Convolutional Transformer
CNN	Convolutional Neural Network
FPS	Frames Per Second
GPS	Global Positioning System
GPU	Graphics Processing Unit
GSD	Ground Sampling Distance
INACAL	Instituto Nacional de Calidad (Peruvian National Institute of Quality)
IoU	Intersection over Union
IR	Imbalance Ratio
ITS	Intelligent Transportation Systems
MAE	Mean Absolute Error
mAP	Mean Average Precision
MJPEG	Motion JPEG
MTC	Ministerio de Transportes y Comunicaciones (Ministry of Transport and Communications)
NMS	Non-Maximum Suppression
NMP	Norma Metrológica Peruana (Peruvian Metrological Standard)
OCR	Optical Character Recognition
RMSE	Root Mean Square Error
RTSP	Real-Time Streaming Protocol
SGD	Stochastic Gradient Descent
SUTRAN	Superintendencia de Transporte Terrestre (National Land Transport Superintendence)
UAV	Unmanned Aerial Vehicle
UIT	Unidad Impositiva Tributaria (Peruvian Tax Reference Unit)
UTM	Universal Transverse Mercator
YOLO	You Only Look Once
