# Supplementary material for: Real-Time Urban Traffic Monitoring System Based on Edge Computing: Vehicle Speed Estimation via Homography and Automatic License Plate Recognition on Jetson Orin NX for Andean Urban Corridors
Source: Sensors (Basel). 2026 Oct 29;26(21):6845. doi: 10.3390/s26216845 (PMC13765829; doi:10.3390/s26216845)
Supplement: Supplementary file 1 [file sensors-26-06845-s001.zip › Supplementary_Material_File S1.pdf]

Accompanying manuscript: "Real-Time Urban Traffic Monitoring System Based on Edge Computing: Vehicle Speed Estimation via Homography and Automatic License Plate Recognition on Jetson Orin NX for Andean Urban Corridors." This document reports, in full, the two validation campaigns (6 July 2026 and 15 August 2026) that are superseded by the corrected-pipeline campaign of 18–19 September 2026 reported in the main text (Section 4.3). The main text retains only a brief summary of the two deployment failure modes diagnosed during these campaigns (RTSP resolution renegotiation and the speed-display defect); this document preserves the complete pass-by-pass statistical detail for completeness and reproducibility.

Supplementary Material S1: Preliminary and Extended Nocturnal Speed Validation Campaigns (Superseded)

Preliminary Validation (6 July 2026)

To validate the accuracy of the homography-based speed estimation, GPS speed measurements were collected using a mobile phone GPS speedometer application (VelocX) during nighttime field testing on 6 July 2026 (20:50–21:00), providing an independent reference of vehicle speed at three synchronized time-points. Simultaneous frames were captured from the system's live monitoring dashboard, showing the estimated speed and tracking ID for the vehicle carrying the GPS device.

Table 1 presents the three validation measurements. The mean absolute error (MAE) is 0.33 km/h and the root mean square error (RMSE) is 0.58 km/h across the three points, with individual errors of 1, 0, and 0 km/h. Speed range covered was 0–14 km/h, corresponding to the observed low-speed traffic conditions at the site during the validation window.

Table 1: GPS-based speed validation. Three synchronized measurements were captured at nighttime on 6 July 2026 with vehicles carrying the GPS reference device. The system estimates were read from the live monitoring dashboard.

Point	Timestamp	GPS (km/h)	System (km/h)	Vehicle ID	Error (km/h)
1	20:51:42	12	11	4411 (A)	1
2	20:51:44	14	14	4411 (A)	0
3	20:57:00	0	0	5357	0
**MAE**					**0.33 km/h**
**RMSE**					**0.58 km/h**

Figure 1 presents the visual evidence of the three validation points, showing the simultaneous GPS reading and the system estimation. The vehicle ID 4411 (automobile class) provided two consecutive readings while advancing through the street, with an acceleration between Points 1 and 2 (12 $\rightarrow$ 14 km/h corresponds to the GPS reading; the system captured both values accurately). Point 3 documents the stopped-traffic case, in which vehicle ID 5357 was among 17 vehicles detected simultaneously by the system, all correctly registering 0 km/h.

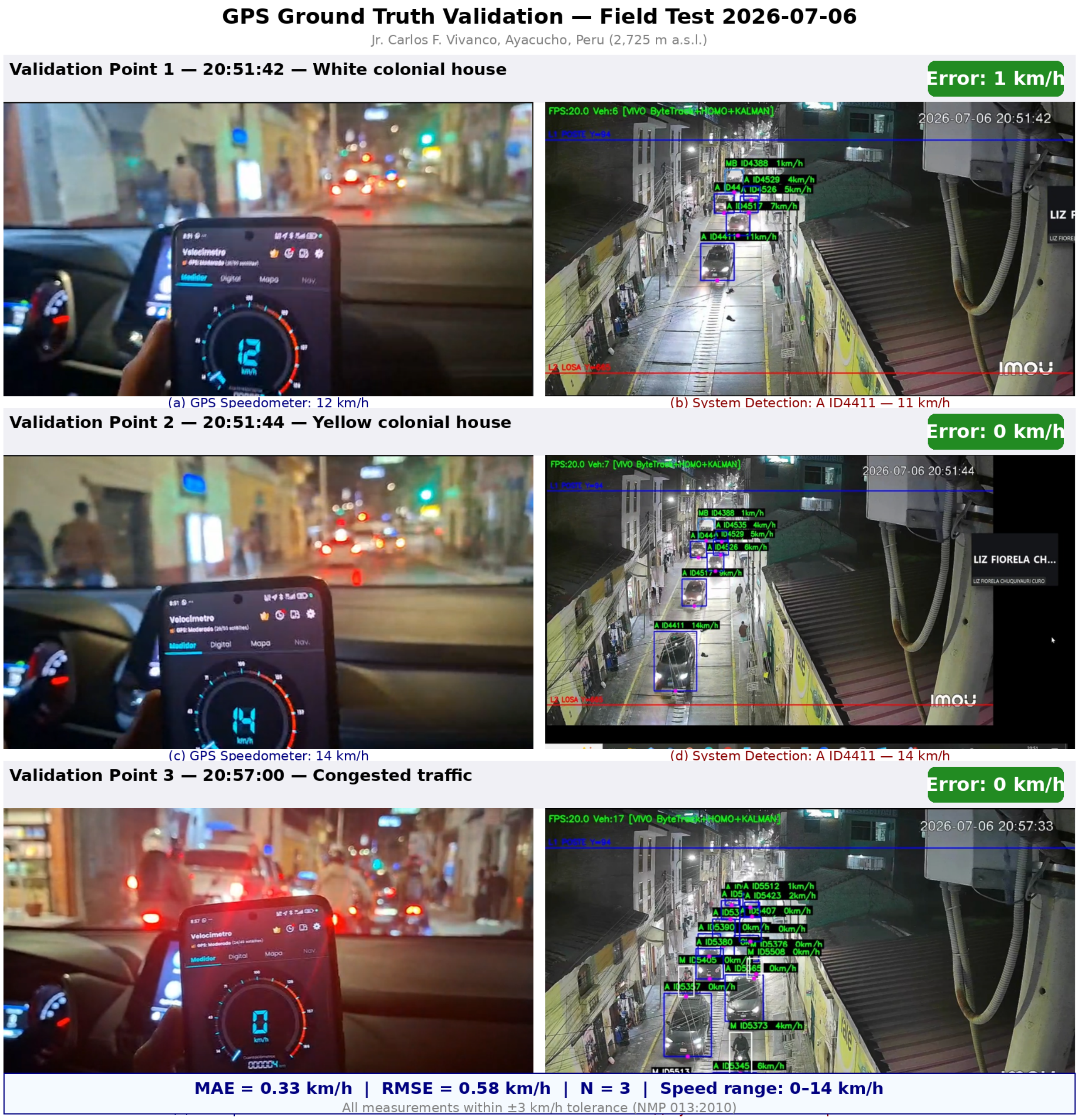

Figure 1: Speed validation against GPS reference. Each validation point shows the simultaneous GPS speedometer reading (left panels) and the system detection with estimated speed (right panels). Point 1 (20:51:42): GPS 12 km/h vs. system 11 km/h (error 1 km/h), class-A vehicle with tracking ID 4411. Point 2 (20:51:44): GPS 14 km/h vs. system 14 km/h (error 0 km/h), the same vehicle ID 4411 advancing along the street (7 vehicles tracked simultaneously). Point 3 (20:57:00): stopped traffic with 17 vehicles detected, vehicle ID 5357 registering 0 km/h. MAE = 0.33 km/h, RMSE = 0.58 km/h, $N = 3$.

Two important qualifications must accompany the interpretation of these results. First, the validation was limited to the 0–14 km/h range due to the observed traffic conditions during the night-time testing window; extended validation covering the enforcement-relevant range (30–60 km/h) is

planned as future work. Second, the sample size ($N = 3$) is small; the reported metrics should be interpreted as a preliminary indication of system accuracy rather than as a definitive characterization. Nevertheless, the consistency of the errors (all ≤ 1 km/h) provides an initial evidence that the homography calibration and the Kalman filtering effectively translate to accurate speed estimates in the operational deployment. This preliminary result, based on only three measurement points from a single vehicle at low speed (0–14 km/h), motivated the extended campaign described below.

Extended Nocturnal Validation Campaign (15 August 2026)

A 24-pass nocturnal validation campaign was conducted on 15 August 2026 (22:12–23:28 local time) using two independent references alongside the proposed system's own output: the vehicle dashboard speedometer, recorded via a GPS Map Camera application overlaying speed on video, and GNSS Doppler velocity recorded at 1 Hz with a BasicAirData GPS Logger. The system's estimated speed was captured via OBS screen recording of the live monitoring dashboard, which began recording at 22:33:07, after the first four passes. The campaign covered a speed range of 0–46 km/h as measured by GPS Doppler (maximum observed in pass 22), encompassing both the 30 km/h legal limit on Jr. Carlos F. Vivanco and the 35 km/h camera tolerance threshold under DS 011-2026-MTC.

Passes with verified calibration integrity (Group A, $N = 2$). Two passes (5 and 6) were captured before an undetected camera resolution change (described below) and are free of the anomalies discussed in this section. Table 2 compares the system's peak estimated speed against the GPS Doppler peak for these two passes.

Table 2: System peak speed versus GPS Doppler peak speed for the two passes with verified calibration integrity (Group A).

Pass	Time	System (km/h)	GPS Doppler (km/h)	Error (km/h)
5	22:33:44	16	20.8	−4.8
6	22:36:29	19	22.4	−3.4
**Mean error**				−4.1 **km/h**

With $N = 2$, these figures are insufficient to characterize system accuracy statistically; they are reported as the only two OBS-covered passes free of the confound described next, and they are superseded as a validation by the 40-pass campaign reported in the main text (Section 4.3). The direction of the underestimation was correctly anticipated here as filter lag, although with two observations its origin could not be established; the September campaign identifies it specifically as the steady-state lag of the constant-velocity motion model under vehicle acceleration, rather than as a start-up transient of the filter.

Undetected camera resolution change (Group B, passes 7–24). Starting at pass 7 (22:37:49), the IMOU aerial camera's RTSP sub-stream renegotiated from the calibrated 1280 × 720 resolution to a higher-resolution stream. The camera model (IMOU IPC-K7C-H1WE) was directly verified against its companion mobile application on 28 August 2026, confirming exactly two available RTSP sub-stream resolutions: 1280 × 720 ("720P") and 2304 × 1296 ("3MP"), a scale factor of 1.8. Because the deployed inference script did not enforce the calibration resolution before computing the homography, pixel coordinates from the higher-resolution stream were fed directly into $\mathbf{H}$, which was fit for the 1280 × 720 frame. This is expected to produce a systematic underestimation of speed

rather than random noise, since it affects the pixel-to-metric scale globally rather than any single measurement.

This was confirmed empirically: comparing the system's peak estimated speed against the GPS Doppler peak for the remaining 18 passes (7–24) yields a ratio of 0.475–0.686 (mean 0.548, median 0.550, SD 0.047; Table 3), a large and consistent departure from the Group A ratio of 0.77–0.85. The direction and order of magnitude are consistent with the $1/1.8 = 0.556$ geometric prediction from the 3MP-stream resolution mismatch, which the observed ratio (0.55 ± 0.05) matches closely; the residual discrepancy between the two figures is well within the partial compensation expected from the Kalman filter's smoothing dynamics. (An earlier internal estimate had assumed a 2880×1620 stream, scale factor 2.25, predicting a ratio of 0.444; this was superseded by the direct camera verification reported above, and the corrected $1.8\times/0.556$ figures are used throughout this work.) Given this identified, systematic, non-random source of error, the raw speed values from Group B passes are not used to compute MAE/RMSE in this work; doing so would conflate a specific, now-corrected software defect with genuine measurement uncertainty. The RTSP request URL specifies `subtype=0`; for the Dahua-family camera protocol that IMOU consumer devices follow, this parameter conventionally selects the main (highest-resolution) sub-stream rather than a secondary stream, which would suggest the 1280×720 stream, not the 2304×1296 stream, was the deviation from the requested configuration. This has not been independently confirmed against the camera's own protocol documentation; regardless of the precise stream-selection mechanism, the empirical evidence—correct-scale operation in Group A (passes 5–6) and the systematic $1.8\times$ scale deviation from pass 7 onward—is consistent with an unrequested mid-session stream change, which the deployed mitigation (below) addresses irrespective of its root cause.

Table 3: System-to-GPS peak speed ratio across the campaign, by group.

**Group**	N	**Ratio range**	**Mean (SD)**
A (passes 5–6, verified resolution)	2	0.769–0.848	0.809
B (passes 7–24, resolution mismatch)	18	0.475–0.686	0.548 (0.047)

Speed display anomaly discovered in the field. Independently of the resolution mismatch, manual frame-by-frame review of the OBS recordings identified a second anomaly: in 8 of the 18 Group B passes (44%; passes 7, 8, 11, 13, 15, 17, 21, and 24), the displayed speed failed to decay toward zero when the tracked vehicle came to a complete stop at the traffic signal, instead holding the last non-zero estimate (12–22 km/h) for 20–40 s. A related but distinct anomaly was observed in pass 20, where the displayed speed dropped non-monotonically ($14 \rightarrow 9$ km/h) without an accompanying deceleration in the reference video, consistent with a tracking identity switch. Group A pass 5 shows an extended plateau (13 km/h sustained for 36 s while the vehicle approached the signal), which the authors' contemporaneous review judged to be genuine gradual deceleration.

At the time, we hypothesised that the resolution mismatch was responsible, by amplifying footpoint position noise and thereby inflating the velocity floor reported for a stationary vehicle. **That hypothesis is withdrawn.** The September campaign reported in the main text reproduces the behaviour with the calibration resolution enforced, which rules out the resolution mismatch as its cause, and traces it instead to the display path: when a track leaves the measurement zone the update routine resets the filter and returns the last stored speed together with a false validity flag, and the on-screen annotation did not test that flag. The measurement chain, the logged speeds and the violation decisions were never affected. In light of the identified mechanism we also consider it more likely than not that the Group A pass 5 plateau was the same display defect rather than

genuine deceleration, since the vehicle was approaching the signal and would have crossed the zone boundary; that earlier determination rested on visual review rather than on a quantitative criterion.

Mitigation. Following identification of the resolution-change mechanism, a corrective measure was implemented and verified on the deployed Jetson .27 system: a mandatory resolution check before homography computation, resizing any frame not matching the calibration resolution (1280 × 720) back to it:

```
if frame.shape[1] != 1280 or frame.shape[0] != 720:
  frame = cv2.resize(frame, (1280, 720), interpolation=cv2.INTER_LINEAR)
```

This fix is included in the public repository accompanying this manuscript (`https://github.com/diegotenorio16-tech/vehicular-speed-estimation-edge-computing`, `inference/prueba_homo_jetson_vivo.py`). It has not yet been subjected to its own dedicated field validation campaign; re-validating the corrected pipeline is noted as future work (Section 6).

Independent documentation of pre-recording passes (1–4). Passes 1–4 (22:12–22:32) preceded the start of OBS recording and therefore have no system output for comparison. GPS Doppler and dashboard speedometer data are nonetheless reported to document the attempted speed range for these passes: 6.7–23.9 km/h (GPS Doppler peak per pass), consistent with the low-speed conditions described in the speedometer log for this portion of the campaign.
