# Supplementary material for: Real-Time Urban Traffic Monitoring System Based on Edge Computing: Vehicle Speed Estimation via Homography and Automatic License Plate Recognition on Jetson Orin NX for Andean Urban Corridors
Source: Sensors (Basel). 2026 Oct 29;26(21):6845. doi: 10.3390/s26216845 (PMC13765829; doi:10.3390/s26216845)
Supplement: Supplementary file 1 [file sensors-26-06845-s001.zip › Supplementary_Material_File S2.pdf]

Accompanying manuscript: "Real-Time Urban Traffic Monitoring System Based on Edge Computing: Vehicle Speed Estimation via Homography and Automatic License Plate Recognition on Jetson Orin NX for Andean Urban Corridors." This document reports, in full, the per-model training configuration of the four segmentation architectures (M1–M4) that were evaluated and ultimately superseded by the detection-only models (M5–M6) deployed on the edge hardware. The main text retains the comparative performance table (Table 3) and the discussion of the methodological transition from segmentation to detection (Section 3.3.1), since those are the results the manuscript's negative-result contribution rests on; this document preserves the individual per-architecture training detail for completeness and reproducibility.

Supplementary Material S2: Per-Architecture Training Configuration of the Segmentation Models (M1–M4, Superseded)

Four segmentation models were trained on the aerial dataset to establish a multi-architecture comparison and identify the optimal architecture for the target deployment. Their comparative performance against the final detection models (M5–M6) is reported in Table 3 of the main text; the per-architecture training configuration is reported here in full.

M1 (YOLOv8m-seg): the 2023-generation medium-sized architecture, serving as the baseline. Trained at 640 px for 300 epochs with early stopping at epoch 124.

M2 (YOLO11m-seg): the 2024-generation medium architecture with architectural improvements over YOLOv8. Trained at 640 px with early stopping at epoch 123.

M3 (YOLO26m-seg): the 2025-generation architecture incorporating NMS-free inference, removal of Distribution Focal Loss (eliminating fixed bounding-box regression ranges), ProgLoss with Small-Target-Aware Label Assignment, and the MuSGD optimizer. Trained at 640 px with early stopping at epoch 204.

M4 (YOLO26m-seg, three-phase progressive): the same YOLO26m architecture trained with a progressive resolution strategy: Phase 1 at 640 px (240 epochs), Phase 2 at 800 px initialized from Phase 1 best weights (77 epochs), and Phase 3 at 1024 px initialized from Phase 2 best weights (21 epochs). Each subsequent phase inherits the learned representations from the previous phase while adapting to finer spatial details at higher resolution. The early convergence in Phases 2 and 3 (best epoch at 4 and 1, respectively) reflects the expected behavior of fine-tuning from a well-initialized checkpoint, rather than premature stopping.

A critical finding during M1 and M2 training was the identification of hyperparameter configurations that caused segmentation mask collapse: `close_mosaic=10`, `mixup=0.1`, `copy_paste=0.1`, `lrf=0.01`, and `amp=True`. These were corrected in M3 and M4 to `close_mosaic=50`, `mixup=0.0`, `copy_paste=0.0`, `lrf=0.001`, and `amp=False`, resolving the mask degradation and producing substantial improvements in segmentation metrics.
